# Global burden of non-communicable chronic diseases associated with a diet low in fruits from 1990 to 2019

**DOI:** 10.3389/fnut.2023.1202763

**Published:** 2023-08-24

**Authors:** Shijie Pan, Zhihan Lin, Teng Yao, Xiaoli Guo, Tongtong Xu, Xinyan Sheng, Xi Song, Zuhai Chen, Wanting Wei, Yizhong Yan, Yunhua Hu

**Affiliations:** ^1^Department of Stomatology, School of Medicine, Shihezi University, Shihezi, Xinjiang, China; ^2^Department of Preventive Medicine, School of Medicine, Shihezi University, Shihezi, Xinjiang, China; ^3^Key Laboratory for Prevention and Control of Emerging Infectious Diseases and Public Health Security, The Xinjiang Production and Construction Corps, Shihezi, Xinjiang, China; ^4^Key Laboratory of Xinjiang Endemic and Ethnic Diseases (Ministry of Education), School of Medicine, Shihezi University, Shihezi, Xinjiang, China; ^5^Key Laboratory of Preventive Medicine, Shihezi University, Shihezi, Xinjiang, China

**Keywords:** global burden, diet low in fruits, epidemiology, disability-adjusted life years, mortality

## Abstract

**Background:**

The aim of this study was to assess the global burden of disease from non-communicable chronic diseases (NCD) due to diet low in fruits from 1990 to 2019.

**Methods:**

Based on data from the Global Burden of Disease (GBD) 2019, the global burden of disease due to diet low in fruits was analyzed for each country or region, disaggregated by disease type, age, sex, and year. The number of deaths and disability-adjusted life years (DALYs), population attributable fraction (PAF), age-standardized mortality rate (ASMR) and age-standardized DALY rate (ASDR) were calculated, and the average annual percentage change (AAPC) was calculated to describe trends in ASMR and ASDR from 1990 to 2019.

**Results:**

From 1990 to 2019, the number of deaths and DALYs due to diet low in fruits increased by 31.5 and 27.4%, respectively. Among the tertiary diseases, ischemic heart disease, stroke, and diabetes and kidney disease were the top three contributors to the global increase in deaths and DALYs. However, both ASMR and ASDR showed a decreasing trend. The fastest decline in ASMR and ASDR was in stroke, with AAPC of −2.13 (95% CI: −2.22, −2.05, *p* < 0.05) and −0.56 (95% CI: −0.62, −0.51, *p* < 0.05), respectively. For GBD regions, high PAF occurred mainly in South Asia, Oceania, and sub-Saharan Africa. Age-specific PAF for stroke and ischemic heart disease death attributable to diet low in fruits was significantly negatively associated with age. Diet low in fruits related ASMR and ASDR showed an M-shaped relationship with the socio-demographic index (SDI), but with an overall decreasing trend.

**Conclusion:**

The number of deaths and DALYs due to diet low in fruits continues to increase. Therefore, early nutritional interventions should be implemented by the relevant authorities to reduce the burden of diseases caused by diet low in fruits.

## 1. Introduction

Dietary nutrition and health are closely related, and deficiencies in nutrients required to maintain general health, such as protein, carbohydrates, lipids, vitamins and minerals, can affect human health (1). Suboptimal diets can lead to deficiencies of these nutrients and thus increase the disease burden of non-communicable chronic diseases (NCD) in humans (2, 3). The suboptimal diets are mainly due to several poor dietary habits, and diet low in fruits is one of the important ones (2). Diet low in fruits was defined as consuming on average less than 250 g of fruit (fresh, frozen, cooked, canned or dried, but excluding fruit juices and preserved fruits) per day (2). Studies have shown that diet low in fruits is associated with diseases such as lung cancer, esophageal cancer, ischemic heart disease, stroke and diabetes (4–6).

**Figure 1:**
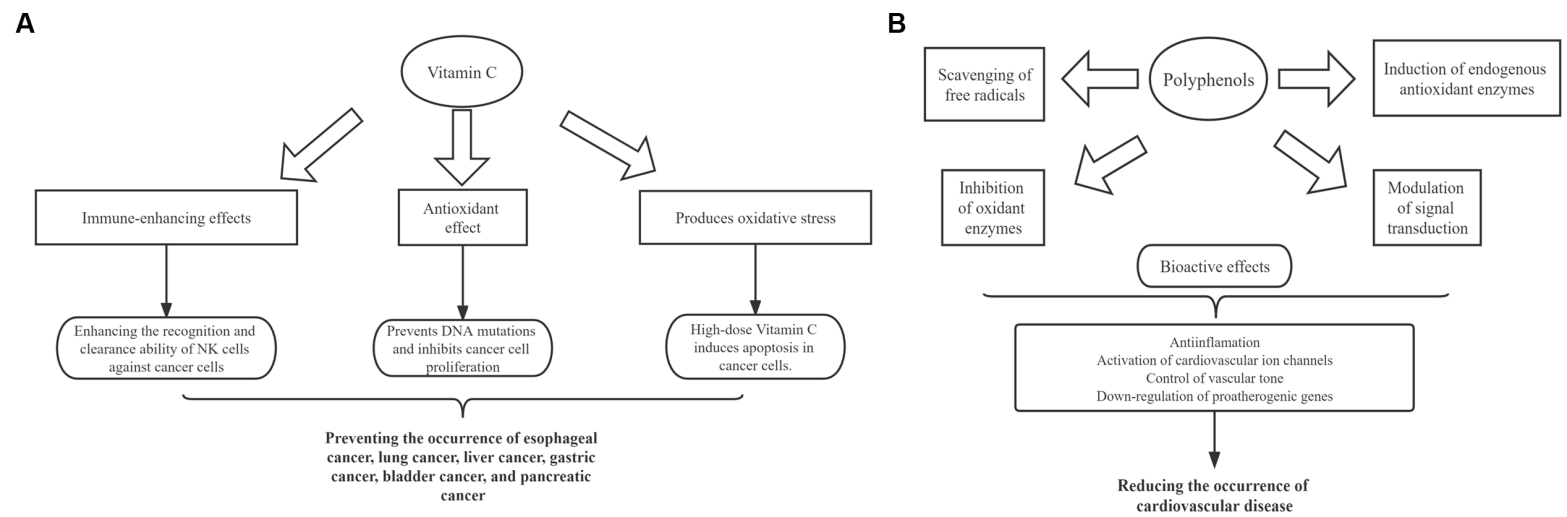
Mechanisms of vitamin C and polyphenols in fruits after entering the body. **(A)** Diagram of the role of fruit vitamin C in cancer prevention. **(B)** Schematic representation of the biological activity of fruit polyphenols in the prevention of cardiovascular diseases.

Fruits contain nutrients that benefit human health and are an important part of a healthy diet. Studies have shown that vitamin C in fruits can destroy cancer cells and inhibit tumor growth, so humans can supplement vitamin C and thus reduce cancer risk by consuming appropriate amounts of fruits (7–10) (Figure 1A). In addition, secondary metabolites in fruits, such as flavonoids, alkaloids, carotenoids, essential oils and phenol acids, show antimutagenic and antiproliferative by inhibiting oxidation, protecting DNA from damage and stimulating tumor cell apoptosis (11, 12). Moreover, fruits play an important role in preventing cardio-vascular diseases (13). It has been found that fruit intake shows a negative correlation with cardiovascular burden (14–17). Fruits are one of the main dietary sources of polyphenols (18), which have a high antioxidant capacity and free radical scavenging ability and can inhibit the production of reactive oxygen species, thereby reducing the occurrence of cardiovascular disease (19) (Figure 1B). In addition, high fruit intake can reduce the risk of diabetes by affecting the metabolism and diversity of the gut microbiota (20–24). Diet low in fruits is closely associated with tumors, cardiovascular diseases and diabetes.

Diet low in fruits is receiving increasing attention worldwide as important dietary risk factors. However, the burden of NCD attributable to diet low in fruits has not been systematically and quantitatively assessed. In this study, we counted the age-standardized mortality rate (ASMR) and age-standardized rate of DALY (ASDR) for diseases attributable to diet low in fruits in 204 countries and territories from 1990 to 2019. The mean annual percentage change (AAPC) of ASMR and ASDR was also calculated using a log-linear regression model to analyze trends from 1990 to 2019. And Pearson correlation coefficient was used to explore the relationship between socio-demographic index (SDI) and ASMR, ASDR attributable to diet low in fruits. This study provides a foundation for developing effective health strategies for dietary habits to reduce the future burden of disease attributable to diet low in fruits in different regions.

## 2. Materials and methods

### 2.1. Study data

Diet low in fruits is one of the risk factors in the Global Burden of Disease (GBD) Study 2019 database (25). The GBD database provides a suitable data source for consistent comparisons of disease burden by age and sex across different locations from 1990 to 2019, including standard epidemiological measures of mortality and health measures such as disability-adjusted life years (DALYs) in disease burden.

Annual cases and corresponding age-standardized rate and a population-attributable fraction (PAF) of disease burden due to diet low in fruits by disease, sex, age, and location from 1990 to 2019 were collected from the Global Health Data Exchange query tool,^1^ a tool that is the most recent estimate of the world’s epidemiological data to provide a comparative assessment of the burden of disease for 209 diseases and injuries and 87 risk factors from 1990 to 2019-divided into 15 age groups of 25–29 years, 30–34 years, 35–40 years to 95 years and older in 5-year intervals. The diseases attributable to diet low in fruits identified in GBD 2019 include esophageal cancer, lung cancer, ischemic heart disease, stroke, and diabetes and kidney diseases. Data on epidemiological and geographic conditions from 204 countries and territories, five SDIs (acting as a composite indicator of developmental status strongly associated with health outcomes, including levels of low, low-middle, middle, high-middle, and high), and 21 GBD regions were available and were used to estimate the burden of disease by locations.

### 2.2. Statistical analysis

To assess the burden of disease and its trends during a specific period, we calculated ASMR/ASDR for mortality and DALYs, and their corresponding AAPC. ASMR/ASDR was calculated by aggregating measures of the rate that a population would have if it had a standard age structure. The weights are taken from the overall distribution of the standard population. The expression was:


$$\mathrm{ASMR}/\mathrm{ASDR}=\frac{\sum_{i=1}^{A}\alpha \mathrm{iwi}}{\sum_{i=1}^{A}\mathrm{wi}}\times 100,000$$

αi: specific age ratio, wi: number (or weight) of selected standard population. Estimated value was used to describe the burden of disease due to diet low in fruits by age, sex and year. To avoid effects due to the age composition of the populations, we chose the age distribution of the world population from the GBD 2019 study to standardize mortality and DALYs per 100,000 person-years for diseases caused by diet low in fruits.

Based on a log-linear regression model, AAPC was calculated to describe the overall time trend in disease ASMR/ASDR. In the model, $In\left(R\right)=a+\beta •T+\varepsilon$, where R is the count or rate, *T* is calendar year, a is the constant term, *β* is the regression coefficient, and ε is the random error term. If both the AAPC and the lower limit of 95% CI were higher than 0, the index was considered to have an upward trend. If both the AAPC and the upper limit of 95% CI were less than 0, the estimated was considered to have a downward trend. Otherwise, the index was considered stable over time (26). Log-linear regression model was applied to analyze the temporal trends and AAPC of diseases attributable to diet low in fruits globally. In addition, the Pearson model was used to explore the relationship between SDI and ASMR and between SDI and ASDR for diseases attributed to diet low in fruits.

## 3. Results

### 3.1. Global burden of disease caused by diet low in fruits in 2019

Globally, the number of deaths caused by diet low in fruits was 1046.01 thousand (95% UI: 730.09, 1363.86), 459.33 thousand (95% UI: 317.06, 601.72) for female and 586.69 thousand (95% UI: 402.51, 777.78) for male (Table 1). The number of DALYs caused by diet low in fruits was 2767.83 thousand (95% UI: 2022.67, 3592.54), 1128.98 thousand (95% UI: 825.67, 1460.12) and 1638.85 thousand (95% UI: 1178.77, 2156.14) for female and male, respectively (Supplementary Table S1).

**Table 1:** Global deaths attributable to diet low in fruits in 1990 and 2019, and the temporal trend from 1990 to 2019.

Cause of deaths	1990			2019			1990–2019	
	Deaths No. × 10^3^ (95% UI)	ASMR per 100,000 (95%UI)	Age-standardized PAF, % (95%UI)	Deaths No. × 10^3^ (95% UI)	ASMR per 100,000 (95%UI)	Age-standardized PAF, % (95%UI)	AAPC of ASMR (95%CI)	AAPC of Age-standardized PAF (95%CI)
**All causes**								
Both	795.57 (560.62, 1034.79)	21.61 (14.98, 28.33)	1.94 (1.34, 2.53)	1046.01 (730.09, 1363.86)	13.12 (9.09, 17.09)	1.79 (1.23, 2.32)	−1.70 (−1.83, −1.57)	−0.28 (−0.38, −0.18)
Female	360.96 (251.35, 473.79)	17.97 (12.44, 23.78)	1.87 (1.31, 2.47)	459.33 (317.06, 601.72)	10.51 (7.25, 13.77)	1.71 (1.19, 2.22)	−1.83 (−1.93, −1.73)	−0.32 (−0.42, −0.21)
Male	434.64 (306.27, 568.38)	25.79 (17.94, 33.76)	1.98 (1.37, 2.57)	586.69 (402.51, 777.78)	16.07 (10.99, 21.38)	1.84 (1.26, 2.41)	−1.62 (−1.69, −1.53)	−0.24 (−0.35, −0.14)
**Disease type**								
**Neoplasms**								
Both	100.25 (56.25, 150.31)	2.56 (1.43, 3.83)	1.73 (0.96, 2.58)	128.38 (65.04, 200.43)	1.58 (0.79, 2.46)	1.26 (0.63, 1.95)	−1.67 (−1.78, −1.55)	−1.10 (−1.15, −1.04)
Female	30.84 (16.89, 47.14)	1.46 (0.79, 2.24)	1.24 (0.68, 1.91)	40.44 (21.15, 59.96)	0.92 (0.48, 1.37)	0.92 (0.48, 1.36)	−1.58 (−1.68, −1.47)	−1.04 (−1.12, −0.95)
Male	69.41 (38.87, 104.54)	3.88 (2.15, 5.78)	2.06 (1.14, 3.11)	87.96 (43.14, 141.81)	2.35 (1.16, 3.78)	1.49 (0.74, 2.36)	−1.73 (−1.94, −1.51)	−1.11 (−1.17, −1.04)
**Esophageal cancer**								
Both	51.87 (17.82, 92.69)	1.32 (0.45, 2.37)	16.14 (5.61, 28.51)	51.21 (15.23, 108.73)	0.63 (0.19, 1.33)	10.27 (3.12, 22.18)	−2.57 (−2.73, −2.41)	−1.54 (−1.59, −1.48)
Female	18.01 (6.63, 32.14)	0.86 (0.31, 1.53)	17.07 (6.34, 29.43)	15.51 (5.19, 30.42)	0.35 (0.12, 0.65)	11.77 (4.06, 23.12)	−3.03 (−3.20, −2.86)	−1.28 (−1.38, −1.18)
Male	33.86 (11.02, 61.13)	1.86 (0.58, 3.39)	15.63 (5.19, 28.01)	35.66 (10.02, 78.76)	0.94 (0.26, 2.09)	9.77 (2.76, 21.98)	−2.36 (−2.60, −2.12)	−1.61 (−1.67, −1.56)
**Tracheal, bronchus, and lung cancer**								
Both	48.39 (15.86, 71.84)	1.24 (0.39, 1.83)	4.53 (1.47, 6.72)	77.19 (22.55, 115.14)	0.95 (0.28, 1.42)	3.78 (1.09, 5.61)	−0.89 (−1.01, −0.78)	−0.62 (−0.64, −0.60)
Female	12.83 (4.16, 19.21)	0.61 (0.18, 0.91)	4.67 (1.54, 6.94)	24.93 (7.19, 37.41)	0.57 (0.16, 0.85)	3.78 (1.09, 5.66)	−0.23 (−0.29, −0.18)	−0.71 (−0.73, −0.69)
Male	35.55 (11.61, 53.11)	2.01 (0.65, 2.97)	4.47 (1.44, 6.62)	52.26 (15.19, 78.62)	1.41 (0.41, 2.12)	3.77 (1.09, 5.63)	−1.21 (−1.38, −1.04)	−0.58 (−0.61, −0.56)
**Cardiovascular diseases**								
Both	654.58 (423.35, 897.38)	17.95 (11.58, 24.61)	5.06 (3.28, 6.85)	829.27 (514.63, 1140.66)	10.44 (6.46, 14.41)	4.35 (2.71, 5.93)	−1.84 (−1.98, −1.70)	−0.52 (−0.55, −0.49)
Female	308.28 (204.08, 421.79)	15.44 (10.05, 21.18)	4.91 (3.23, 6.61)	373.39 (230.74, 510.97)	8.54 (5.29, 11.69)	4.19 (2.63, 5.68)	−2.01 (−2.15, −1.87)	−0.54 (−0.58, −0.51)
Male	346.27 (221.36, 479.41)	20.76 (13.16, 28.76)	5.16 (3.34, 7.03)	455.88 (281.51, 635.26)	12.53 (7.71, 17.43)	4.46 (2.76, 6.13)	−1.72 (−1.82, −1.62)	−0.50 (−0.54, −0.46)
**Ischemic heart disease**								
Both	310.72 (129.32, 458.43)	8.77 (3.66, 12.95)	5.15 (2.15, 7.56)	436.45 (179.36, 659.26)	5.53 (2.28, 8.37)	4.69 (1.91, 6.94)	−1.57 (−1.73, −1.41)	−0.32 (−0.39, −0.25)
Female	137.88 (57.08, 202.43)	7.11 (2.96, 10.49)	5.01 (2.06, 7.35)	189.35 (76.67, 286.58)	4.33 (1.76, 6.55)	4.55 (1.86, 6.76)	−1.68 (−1.85, −1.52)	−0.33 (−0.36, −0.30)
Male	172.84 (72.42, 255.08)	10.64 (4.44, 15.79)	5.18 (2.16, 7.63)	247.11 (102.19, 371.98)	6.86 (2.77, 10.33)	4.75 (1.92, 7.05)	−1.48 (−1.67, −1.29)	−0.31 (−0.39, −0.22)
**Stroke**								
Both	343.86 (206.87, 528.73)	9.18 (5.44, 14.12)	6.93 (4.16, 10.52)	392.82 (228.22, 604.23)	4.91 (2.84, 7.57)	5.83 (3.43, 8.95)	−2.13 (−2.22, −2.05)	−0.59 (−0.62, −0.56)
Female	170.39 (100.66, 261.82)	8.33 (4.86, 12.85)	6.78 (4.06, 10.31)	184.04 (106.59, 283.78)	4.21 (2.44, 6.49)	5.73 (3.34, 8.65)	−2.32 (−2.42, −2.22)	−0.57 (−0.59, −0.54)
Male	173.47 (103.39, 265.92)	10.12 (5.97, 15.68)	7.06 (4.25, 10.75)	208.77 (121.28, 324.93)	5.67 (3.26, 8.83)	5.88 (3.42, 9.06)	−1.97 (−2.06, −1.88)	−0.63 (−0.69, −0.57)
**Diabetes and kidney diseases**								
Both	40.77 (25.66, 56.78)	1.08 (0.69, 1.54)	3.21 (2.02, 4.53)	88.34 (56.09, 126.27)	1.09 (0.7, 1.58)	2.92 (1.81, 4.19)	0.02 (−0.13, 0.16)	−0.34 (−0.42, −0.25)
Female	21.84 (13.34, 31.39)	1.07 (0.65, 1.53)	3.34 (2.07, 4.75)	45.45 (27.92, 65.14)	1.04 (0.64, 1.49)	3.04 (1.88, 4.39)	−0.09 (−0.23, 0.06)	−0.31 (−0.43, −0.19)
Male	18.92 (12.05, 26.12)	1.16 (0.73, 1.61)	3.04 (1.93, 4.25)	42.84 (26.57, 61.46)	1.19 (0.73, 1.71)	2.78 (1.72, 3.98)	0.08 (−0.11, 0.27)	−0.32 (−0.44, −0.19)
**Diabetes mellitus**								
Both	40.77 (25.66, 56.76)	1.07 (0.69, 1.54)	6.16 (3.89, 8.68)	88.34 (56.09, 126.27)	1.08 (0.69, 1.58)	5.68 (3.47, 8.14)	0.02 (−0.13, 0.16)	−0.28 (−0.43, −0.13)
Female	21.84 (13.34, 31.39)	1.07 (0.65, 1.53)	6.02 (3.77, 8.54)	45.48 (27.92, 65.14)	1.04 (0.64, 1.49)	5.68 (3.55, 8.19)	−0.09 (−0.23, 0.06)	−0.18 (−0.32, −0.05)
Male	18.92 (12.05, 26.12)	1.16 (0.73, 1.61)	6.36 (4.04, 8.87)	42.84 (26.57, 61.46)	1.19 (0.73, 1.71)	5.66 (3.51, 8.13)	0.08 (−0.11, 0.27)	−0.41 (−0.44, −0.37)

ASMR, age-standardized mortality rate; PAF, population attributable fraction; AAPC, average annual percentage change; UI, uncertainty interval; CI, confidence interval.

At the SDI region level, the middle SDI region had the highest number of deaths attributable to diet low in fruits (325.58 thousand) and the highest number of DALYs (876.18 thousand), but the region with the highest ASMR and ASDR were low-middle SDI region. Among the 21 GBD regions, East Asia and South Asia ranked the top two in terms of deaths or DALYs attributable to diet low in fruits, but the top two ASMR occurred in Central Asia and Oceania, and the top two ASDRs occurred in South Asia and Oceania (Supplementary Tables S2, S3).

Among the five tertiary diseases, the top three diseases in terms of the number of mortality and DALYs attributable to diet low in fruits were: ischemic heart disease, stroke, and diabetes and kidney disease, accounting for 87.7 and 89.2% of the total, respectively (Table 1; Supplementary Table S1). In addition, the three diseases mentioned above also ranked in the top three in ASMR and ASDR (Table 1; Supplementary Table S1).

**Figure 2:**
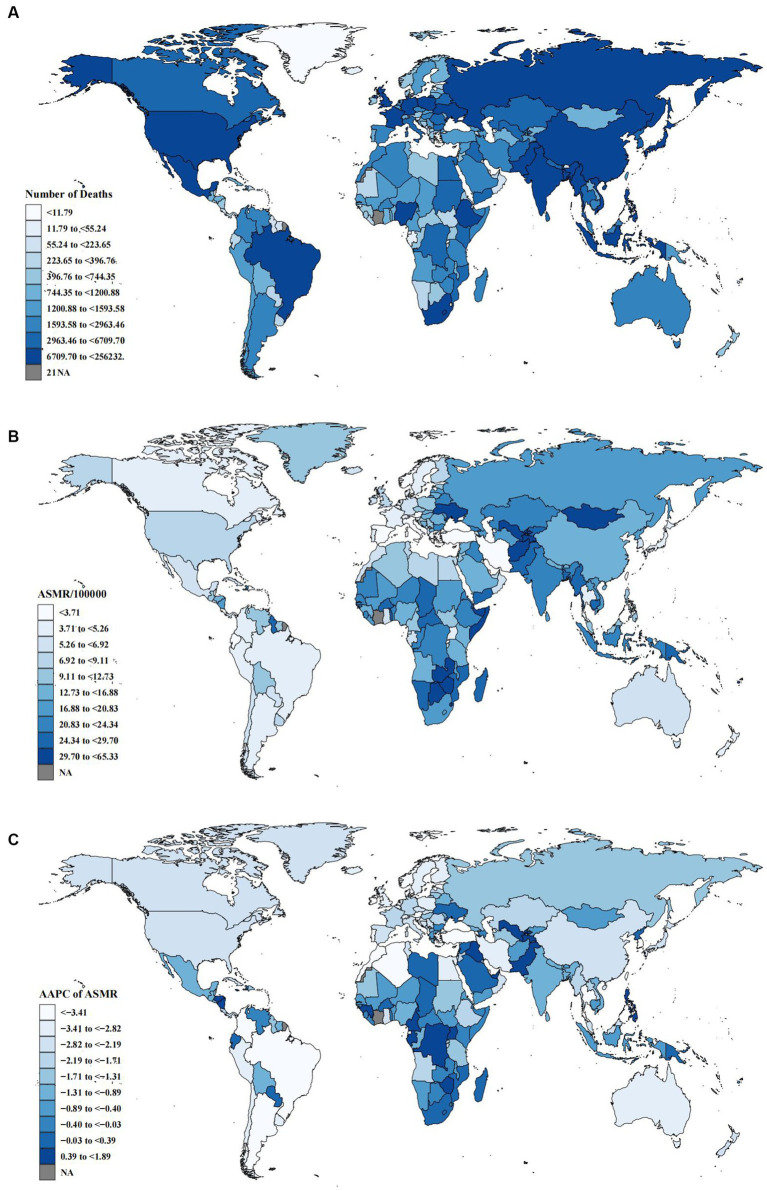
Global deaths burden attributable to diet low in fruits for both sexes. **(A)** Number of deaths in 2019. **(B)** ASMR in 2019. **(C)** AAPC of ASMR from 1990 to 2019.

At the country or territory level, the top three deaths attributable to diet low in fruits were in India, China and Russia (Supplementary Table S4; Figure 2A), while India, China and Indonesia were the top three DALYs attributable to diet low in fruits (Supplementary Table S4; Supplementary Figure S1A). The top three ASMR attributable to diet low in fruits were Solomon Islands, Mongolia and Fiji (Supplementary Table S4; Figure 2B), while Solomon Islands, Kiribati and Fiji were the top three ASDRs attributable to diet low in fruits (Supplementary Table S4; Supplementary Figure S1B).

### 3.2. Trends in the burden of disease attributable to diet low in fruits from 1990 to 2019

The global number of deaths caused by diet low in fruits increased from 795.57 thousand (95% UI: 566.62, 1034.79) in 1990 to 1046.01 thousand (95% UI: 730.09, 1363.86) in 2019, an increase of 31.5%. However, ASMR showed a decreasing trend overall, males and females with AAPC of −1.70 (95% CI: −1.83, −1.57), −1.62 (95% CI: −1.69, −1.53) and −1.83 (95% CI: −1.93, −1.73), respectively (Table 1; Supplementary Figures S2A, S3A). From 1990 to 2019, the DALYs number in-creased by 27.4%, and ASDR decreased slightly with AAPC of −1.56 (95% CI: −1.69, −1.42). Both male and female ASDR dropped with AAPC of −1.49 (95% CI: −1.63, −1.34) and − 1.66 (95% CI: −1.75, −1.58), respectively (Supplementary Table S1; Supplementary Figures S2B, S3B).

At the SDI region level, ASMR and ASDR decreased in all five SDI regions, with the fastest decrease in ASMR in the high SDI region with AAPC of −2.67 (95% CI: −2.85, −2.49) and the fastest decrease in ASDR in the high-middle SDI region with AAPC of −2.29 (95% CI: −2.57, −2.02). Regarding the GBD region, ASMR in Sub-Saharan Africa and ASDR in Oceania were the fastest growing with AAPC of 0.09 (95% CI: −0.48, 0.66) and 0.09 (95% CI: 0.05, 0.13), respectively. The ASMR and ASDR in Southern Latin America were the fastest de-creasing with AAPC of −3.73 (95% CI: −4.01, −3.46) and −3.65 (95% CI: −3.87, −3.43), respectively (Supplementary Tables S2, S3). At the country or territory level, the fastest decrease in ASMR or ASDR was in Singapore, and the most rapid growth in ASMR or ASDR was in the UAE (Figure 2C, Supplementary Table S4; Supplementary Figure S1C).

Among the 5 level-three diseases, ischemic heart disease, stroke, and diabetes and kidney diseases were the top three contributors to the global increase in deaths due to diet low in fruits, contributing 88.6% of the total increase in deaths from 1990 to 2019. ASMR decreased significantly in patients with stroke and ischemic heart disease, with AAPC of −2.13 (95% CI: −2.22, −2.05) and −1.57 (95% CI: −1.73, −1.41), while ASMR did not change much in patients with diabetes and kidney diseases, with AAPC of 0.02 (95% CI: −0.13, 0.16) (Table 1). Similar to the mortality trend, the main contribution of ischemic heart disease, diabetes and kidney diseases, and stroke to the overall increase in DALYs was 42.2, 36.3 and 14.9%, respectively. Among them, ASDR decreased more rapidly in stroke with AAPC of −0.56 (95% CI: −0.62, −0.51) (Supplementary Table S1).

### 3.3. PAF of the diseases attributable to diet low in fruits

**Figure 3:**
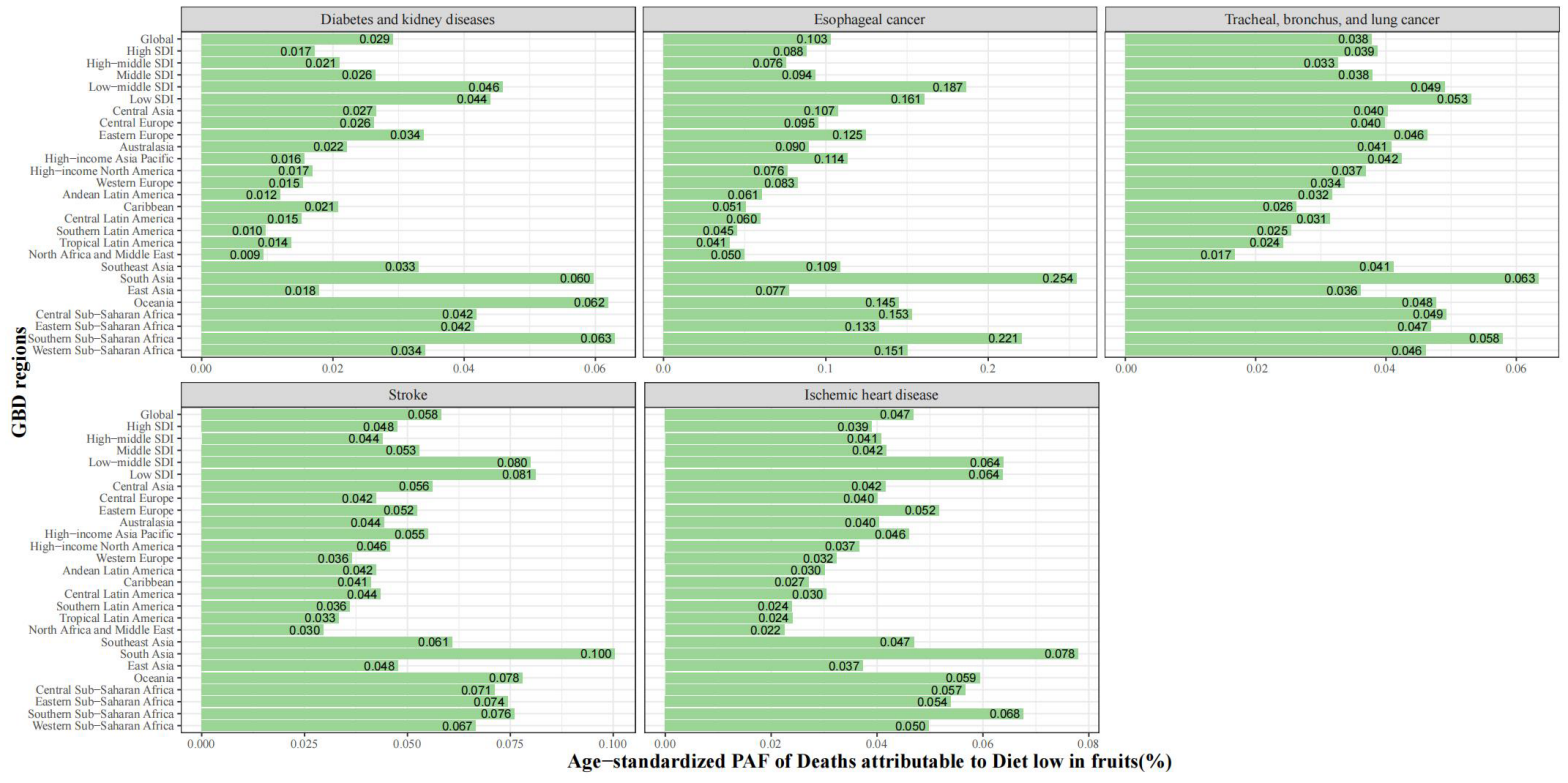
Age-standardized PAF of deaths of specific GBD level-three diseases attributable to diet low in fruits by region for both sexes in 2019.

The PAF of ASMR for five tertiary diseases varied significantly between different regions (Figure 3). In 5 level-three diseases, the top three PAF for esophageal cancer, stroke and ischemic heart disease were 4.7, 5.8, and 10.3%, respectively (Table 1). At the SDI region level, the highest PAF was mainly in low-middle and middle SDI regions. At the GBD region level, the highest PAF was primarily in Eastern Europe, Central Asia and South Asia (Supplementary Table S2).

**Figure 4:**
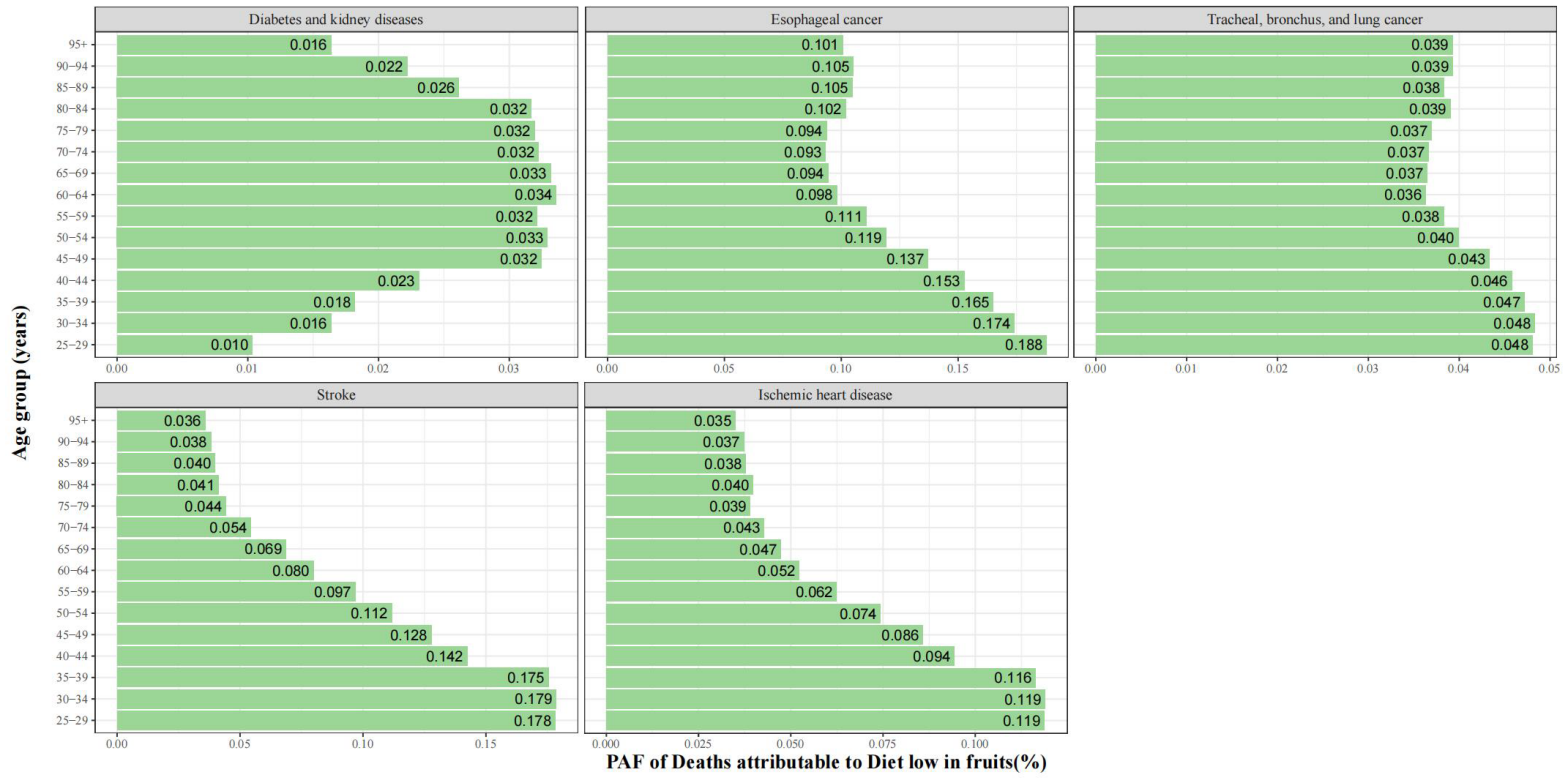
PAF of deaths of specific GBD level-three diseases attributable to diet low in fruits by age group for both sexes in 2019.

Trends in PAF for deaths attributable to diet low in fruits varied with age across the 5 level-three disease types (Figure 4). Age-specific PAF for death from stroke and ischemic heart disease was significantly negatively associated with age. Age-specific PAF for esophageal cancer deaths had a slight negative association with age until 60–64. Age-specific PAF for deaths from diabetes and kidney diseases were slightly elevated before 45–49 years and slightly decreased after 80–84 years. A similar trend was observed for PAF in DALYs (Supplementary Figure S4).

### 3.4. Association of diet low in fruits disease burden with SDI

**Figure 5:**
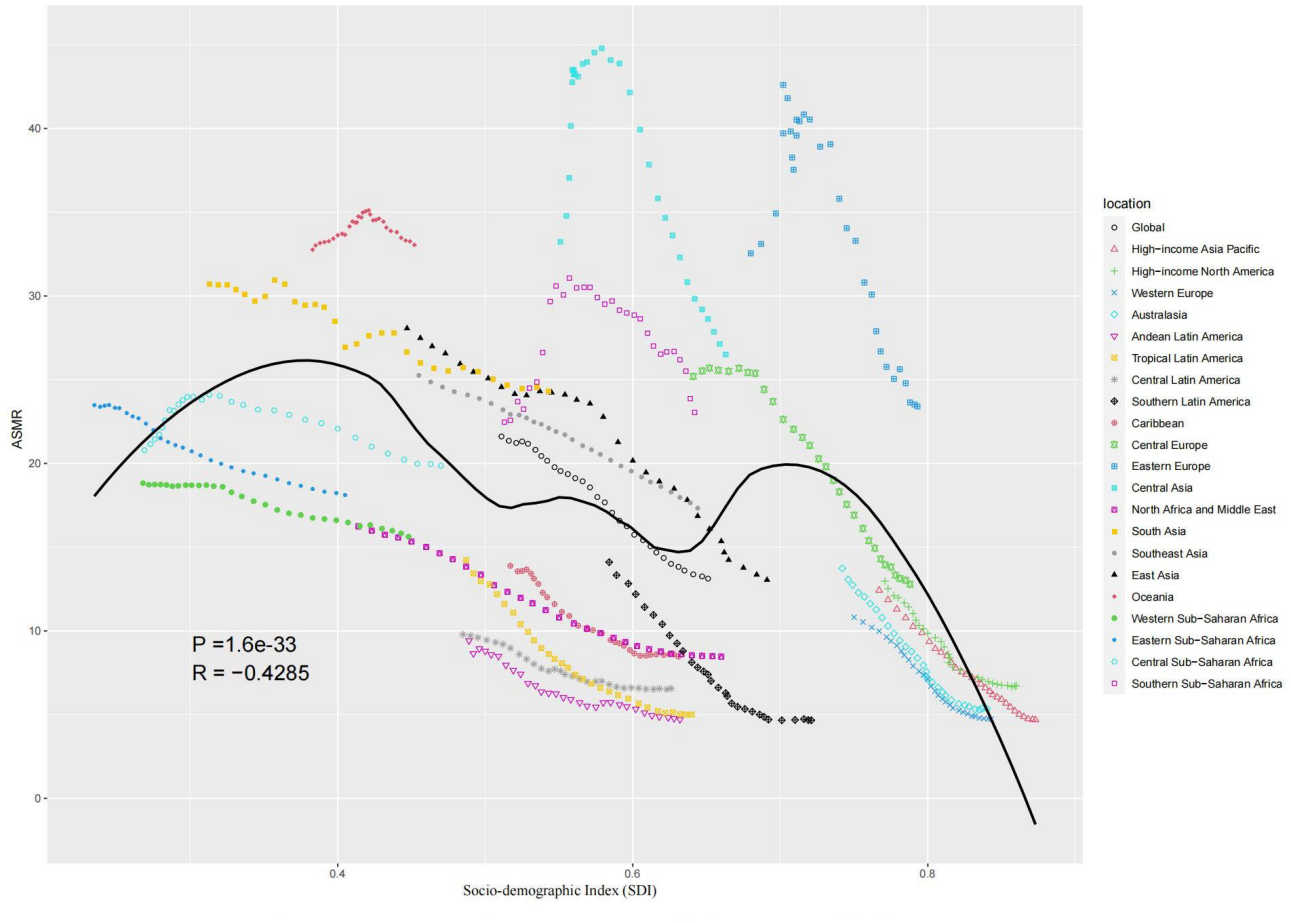
Age-standardized burden rate attributable to diet low in fruits across 21 GBD regions by the socio-demographic index for both sexes, 1990–2019.

**Figure 6:**
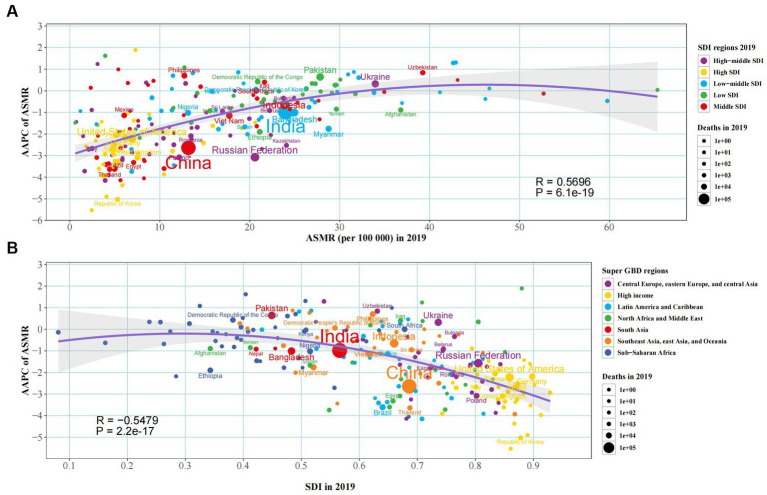
The factors associated with the AAPC of ASMR attributable to diet low in fruits from 1990 to 2019, both sexes, at the national level. **(A)** The corresponding ASMR in 2019; **(B)** Socio-demographic index in 2019.

The ASMR and ASDR showed an overall “M” relationship with SDI, with a negative correlation, reaching the first peak at SDI = 0.4 and the second peak at SDI = 0.7 (Figure 5, Supplementary Figure S5). AAPC of ASMR or ASDR had a weak positive correlation (correlation coefficient around 0.15) (Supplementary Figures S6A, S7A). The AAPC of ASMR had a significant negative correlation in 2019 (correlation coefficient around −0.55), more pronounced when SDI was greater than 0.5 (Figure 6). The AAPC of ASDR had a low negative correlation with SDI in 2019 (correlation coefficient around −0.47), more pronounced when SDI was greater than 0.6 (Supplementary Figure S8B).

## 4. Discussion

Using GBD study 2019 data, we systematically estimated trends in the burden of NCD due to diet low in fruits from 1990 to 2019. Globally, from 1990 to 2019, the number of deaths and DALYs attributed to diet low in fruits in-creased. But ASMR and ASDR decreased slightly, mainly in ischemic heart disease, stroke, and diabetes and kidney disease. Compared to females, the males had a more severe disease burden. The ASMR and ASDR caused by diet low in fruits were more significant in areas with low SDI and high-middle SDI, mainly in South Asia and Eastern Europe. Furthermore, PAF for ASMR or ASDR varied significantly between regions and age groups in the five tertiary diseases. These results help guide public health programs and inform strategies to change eating habits and improve health.

In this study, it was found that the mortality rate and DALYs rate of elderly individuals due to diet low in fruits were higher than those of young individuals. This trend may be attributed to the weakened metabolism of elderly, which makes them less effective at absorbing and utilizing the nutrients in fruits within the timeframe when adverse effects of low fruit intake occur (27). In addition, energy intake varies by age group and physical activity level and may also play a key role in the mechanism that causes the burden difference between elderly and younger adults (28, 29). Therefore, it is necessary to carry out early nutritional intervention for the elderly population. By encouraging elderly to increase the intake of fruits, strengthening nutritional guidance, increasing physical activities and providing suitable dietary services, and other measures to improve the dietary health and metabolic capacity of elderly, and improve nutritional intake of elderly. Thus, the burden of elderly due to diet low in fruits may be reduced.

Between 1990 and 2019, the burden of disease due to diet low in fruits in-creased in both males and females, with age-wide increases in the number of deaths and DALYs, but corresponding ASMR/ASDR decreased. This may result from the rapid growth of the global population (30). This study revealed a significant sex disparity in the burden of cardiovascular disease caused by diet low in fruits. Specifically, the burden was found to be significantly higher in males compared to females in both 1990 and 2019. This is because there are certain differences in the absorption and function of nutrients in fruits in different sexes, leading to varying impacts on their respective bodies. Studies have shown that polyphenols are a kind of natural antioxidant, which can help reduce the production of free radicals and damage to cells, thus having a protective effect on cardiovascular diseases (31). Although polyphenols are beneficial to both sexes, polyphenols have a more beneficial impact on the cardiovascular health of females compared to males (32). Females have better absorption and utilization of polyphenols because they have stronger gastric acid secretion and intestinal absorption ability after eating (33). As a result, the females can better obtain nutrients from polyphenols and thus better protect their cardiovascular system.

Around the world, ASMR or ASDR attributed to diet low in fruits showed an M-shaped association with SDI, showing an overall downward trend, while low SDI and high-middle SDI regions had higher disease burdens than other regions. The causes of death from NCD in low SDI regions include poor living environment, infection, inadequate resources and lack of access to health care (34). The disease burden attributed to diet low in fruits is higher in high-middle SDI areas, which may be due to the traditional diet in the area (35). For example, the conventional diet in Eastern Europe is dominated by meat, cereals and bread. The development of modern technology promotes the consumption of conventional diet categories in Eastern Europe, thus reducing the consumption of fruits and indirectly increasing the burden of cardiovascular diseases in the region (36).

In addition, this study found that the number of deaths and DALYs attributable to diet low in fruits were the largest in middle SDI regions, and the middle SDI regions were represented by countries such as China, Albania and Brazil. Studies have shown that young people in China prefer to eat fruit, consuming about twice as much fruit as elderly (37). China has a large population, and in recent years, the aging degree has intensified (38). This may be an important reason for the above results. All five tertiary diseases in this study had higher PAF for deaths and DALYs in low SDI regions and low-middle SDI regions than in the other three SDI regions. Because of this phenomenon, it is speculated that people in low and low-middle-income countries may lack an understanding of the health benefits of fruits, and the fruits cannot enter the fresh food market due to traffic restrictions (39). It is recommended to increase publicity and access to fruit in low and low-middle-income countries, while increasing the availability and import of fruit. This requires the efforts of governments, businesses and society to encourage people to increase their consumption and intake of fruit through various means to improve the health status of low and low-middle-income countries.

At the regional level of GBD, the highest PAFs of ASMR attributed to diet low in fruits in 2019 were in Eastern Europe, Central Asia and South Asia (2.78, 2.66, and 2.65%). The reason may be the low fruit intake caused by the traditional diet culture in Eastern Europe (15). The *per capita* fruit intake in countries represented by Russia, Ukraine and Belarus in Eastern Europe is less than 300 g/d, while the *per capita* fruit intake recommended by WHO is 400 g/d (40, 41). The street food trade is a well-developed activity in Central Asian cities, often organized in typical markets called bazaars. Street food outlets are common, reflecting the high cultural and dietary importance of street food in the region (42–45). In all the cities surveyed, the fruit was the least common street food (46). The gross national income of South Asian countries is low, and the cost of buying fruit is much higher than that of other economic regions (47). In addition, low-income people are more susceptible to the influence of traditional dietary concepts, and prejudice against the mass consumption of fruits, which may lead to the low intake of fruit in South Asian countries (48). We also found that Oceania had higher ASMR and ASDR, consistent with the previous research (49). The diet quality of Australian residents could be further improved by increasing the consumption of fruits and more types of food.

At the national level, the results showed that China and India were the top two countries regarding the number of deaths and DALYs attributable to diet low in fruits. Data show that in South Asia, China and other regions, higher fruit in-take is negatively correlated with the total mortality risk (50). Therefore, diet low in fruits will lead to a higher deaths and DALYs. At the same time, because China and India are both populous countries, the base is larger. These are the key rea-sons for the above results. We also found that the top ranking of ASMR and ASDR attributable to diet low in fruits were both in the Solomon Islands. Studies have shown that the *per capita* fruit intake in the Solomon Islands has decreased, and the consumption of sugar-sweetened beverages is the lowest, but the prevalence of hypercholesterolemia has increased significantly (51). The reason is that energy comes from the three major productive nutrients of protein, carbohydrates and lipids. The intake of diet low in fruits reduces the dietary fiber in carbohydrates. At the same time, due to the total energy demand, excessive intake of proteins and lipids leads to increased disease burden.

The top three deaths, DALYs, ASMR, and ASDR, attributed to diet low in fruits were ischemic heart disease, stroke, and diabetes and kidney disease. This is consistent with the results of a previous study (52), which found that cardiometabolic death was closely related to suboptimal dietary intake (48.6% in males and 41.8% in females). Diet-related cardiometabolic deaths were the highest, with low fruit accounting for 7.5%. Diet low in fruits is therefore strongly associated with the proportion of deaths from ischemic heart disease, stroke, and diabetes and kidney disease. The reason may be closely related to the functions of various vitamins, minerals and other fruit-rich nutrients (53, 54).

Different countries have formulated various policies and strategies to increase people’s awareness of fruit consumption. For instance, Australia launched the “Go for 2&5” campaign, which was government-funded and utilized multiple media channels to promote a positive attitude toward consuming more fruits and encourage adults to aim for two servings of fruits per day. The Centers for Disease Control and Prevention in the United States has developed the “Fruits & Veggies - More Matters” program. This program encourages people to increase their fruit intake through methods like educational campaigns, diverse promotions, providing tools and support, and establishing partnerships, aiming to promote a healthy lifestyle. In the United Kingdom, there is a program called “Food Dudes” that aims to enhance children’s awareness of fruits through role-playing. This program empowers children with superpowers to fight against villains by tasting various fruits, aiming to inspire them to consume more fruits (55). Due to different levels of economic development in other parts of the world, People’s daily intake of fruit content also varies (41). Population-level dietary interventions are needed to reduce the disease burden associated with diet low in fruits in different regions, especially in areas with low SDI regions. Fruit intake can be increased by raising public awareness of healthy eating through the news media. Improve fruit availability to ensure that people in all regions have access to fresh fruit. More appropriate health strategies are proposed according to dietary habits and disease burden in different regions. These initiatives collectively form global efforts aimed at raising public awareness of healthy eating habits and encouraging people to increase their intake of fruits in innovative and proactive ways.

This study has some limitations. The data comes from the GBD 2019 database, and there may be some differences in data quality and accuracy between different regions and countries. The estimated burden of disease will inevitably be somewhat bias. According to the current data, we are unable to discuss the impact of different types of fruit deficiencies on disease burden. The interrelationship between dietary factors may influence the estimated burden of disease caused by a single dietary component. To address these limitations, we need to design further large-scale epidemiological studies to understand better the burden of NCD caused by diet low in fruits. In addition, intervention studies by providing participants with a variety of fruits are particularly important to explore further the actual size the impact of diet low in fruits on the risk of NCD.

## 5. Conclusion

ASMR and ASDR from diet low in fruits have decreased globally, but the number of deaths and DALYs has still increased significantly from 1990 to 2019. Reducing the nutritional burden caused by diet low in fruits has become a top priority for national and regional governments. It is noteworthy that the burden is significantly higher in the elderly. Ensuring sufficient nutrition for the elderly becomes particularly important due to factors such as declining physical function and eating disorders. It is recommended to take early nutritional intervention measures for the elderly population. Providing a diverse and balanced diet plan, including increased intake of fruits, can help older adults maintain a healthy nutritional status and reduce the risk of diseases. In order to reduce the disease burden caused by diet low in fruits among different age groups, it is suggested to establish appropriate dietary guidelines and implement relevant strategic measures to encourage increased fruit intake. Especially in low SDI and low-middle SDI countries, it is crucial to enhance the dissemination of knowledge about healthy eating. Activities such as public awareness campaigns, training programs for health educators, and providing easily accessible information resources can enhance people’s understanding and awareness of healthy eating, thereby reducing health problems associated with low fruit intake. This requires joint efforts from governments, social organizations, and individuals to promote nutritional health and enhance overall quality of life.

## Data availability statement

The original contributions presented in the study are included in the article/Supplementary material, further inquiries can be directed to the corresponding authors.

## Author contributions

SP, ZL, TY, and YY initiated the study. SP, ZL, TY, XG, TX, and XSh collected and processed the data. WW, XSo, and ZC performed the statistical analysis and visualization. SP and ZL drafted the manuscript. YH, TY, and YY revised the manuscript. The corresponding authors attest that all listed authors meet authorship criteria and that no others who meet these criteria have been omitted. All authors read and approved the final manuscript, contributed to the framework construction, result interpretation, manuscript revision, approved the final version of the manuscript, contributed to the article, and approved the submitted version.

## Funding

This work was supported by Shihezi University High-level Talents Program (RCZK2021B28), the Shihezi University self-funded project (ZZZC202125), the Shihezi University College students’ innovation and entrepreneurship training program (SRP2022084), the Major Public Health Special Project of Xinjiang Production and Construction Corps (20100409), Science and Technology Project of the 8th Division of Xinjiang Production and Construction Corps (2018YL11), the seventh batch of “3152” Young Backbone Teacher project of Shihezi University.

## Conflict of interest

The authors declare that the research was conducted in the absence of any commercial or financial relationships that could be construed as a potential conflict of interest.

## Publisher’s note

All claims expressed in this article are solely those of the authors and do not necessarily represent those of their affiliated organizations, or those of the publisher, the editors and the reviewers. Any product that may be evaluated in this article, or claim that may be made by its manufacturer, is not guaranteed or endorsed by the publisher.

## Footnotes

^1^ https://vizhub.healthdata.org/gbd-results/
