# Corrigendum: Global burden of non-communicable chronic diseases associated with a diet low in fruits from 1990 to 2019

**DOI:** 10.3389/fnut.2023.1285805

**Published:** 2023-09-12

**Authors:** Shijie Pan, Zhihan Lin, Teng Yao, Xiaoli Guo, Tongtong Xu, Xinyan Sheng, Xi Song, Zuhai Chen, Wanting Wei, Yizhong Yan, Yunhua Hu

**Affiliations:** ^1^Department of Stomatology, School of Medicine, Shihezi University, Shihezi, Xinjiang, China; ^2^Department of Preventive Medicine, School of Medicine, Shihezi University, Shihezi, Xinjiang, China; ^3^Key Laboratory for Prevention and Control of Emerging Infectious Diseases and Public Health Security, The Xinjiang Production and Construction Corps, Shihezi, Xinjiang, China; ^4^Key Laboratory of Xinjiang Endemic and Ethnic Diseases (Ministry of Education), School of Medicine, Shihezi University, Shihezi, Xinjiang, China; ^5^Key Laboratory of Preventive Medicine, Shihezi University, Shihezi, Xinjiang, China

**Keywords:** global burden, diet low in fruits, epidemiology, disability-adjusted life years, mortality

In the published article, there was an error in the author list. Author Teng Yao was erroneously excluded. The corrected author list appears below.

Shijie Pan^1†^, Zhihan Lin^1†^, Teng Yao^2†^, Xiaoli Guo^1^, Tongtong Xu^2^, Xinyan Sheng^1^, Xi Song^2^, Zuhai Chen^2^, Wanting Wei^2^, Yizhong Yan^2,3,4,5*^ and Yunhua Hu^2,3,4,5*^

In the published article, there was an error. The Author Contributions section was missing an author.

This sentence previously stated:

“SP, ZL and YY initiated the study. SP, ZL, XG, TX, and XSh collected and processed the data. WW, XSo, and ZC performed the statistical analysis and visualization. SP and ZL drafted the manuscript. YH, and YY revised the manuscript. All authors read and approved the final manuscript. All authors contributed to the framework construction, result interpretation, and manuscript revision. All authors approved the final version of the manuscript. The corresponding authors attest that all listed authors meet authorship criteria and that no others who meet these criteria have been omitted. All authors contributed to the article and approved the submitted version.”

The corrected sentence appears below:

“SP, ZL, TY, and YY initiated the study. SP, ZL, TY, XG, TX, and XSh collected and processed the data. WW, XSo, and ZC performed the statistical analysis and visualization. SP and ZL drafted the manuscript. YH, TY, and YY revised the manuscript. The corresponding authors attest that all listed authors meet authorship criteria and that no others who meet these criteria have been omitted. All authors read and approved the final manuscript, contributed to the framework construction, result interpretation, manuscript revision, approved the final version of the manuscript, contributed to the article, and approved the submitted version.”

The authors apologize for this error and state that this does not change the scientific conclusions of the article in any way. The original article has been updated.

