# Supplementary figures and images for: Global burden of non-communicable chronic diseases associated with a diet low in fruits from 1990 to 2019
Source: Front Nutr. 2023 Aug 24;10:1202763. doi: 10.3389/fnut.2023.1202763 (PMC10491017; doi:10.3389/fnut.2023.1202763)

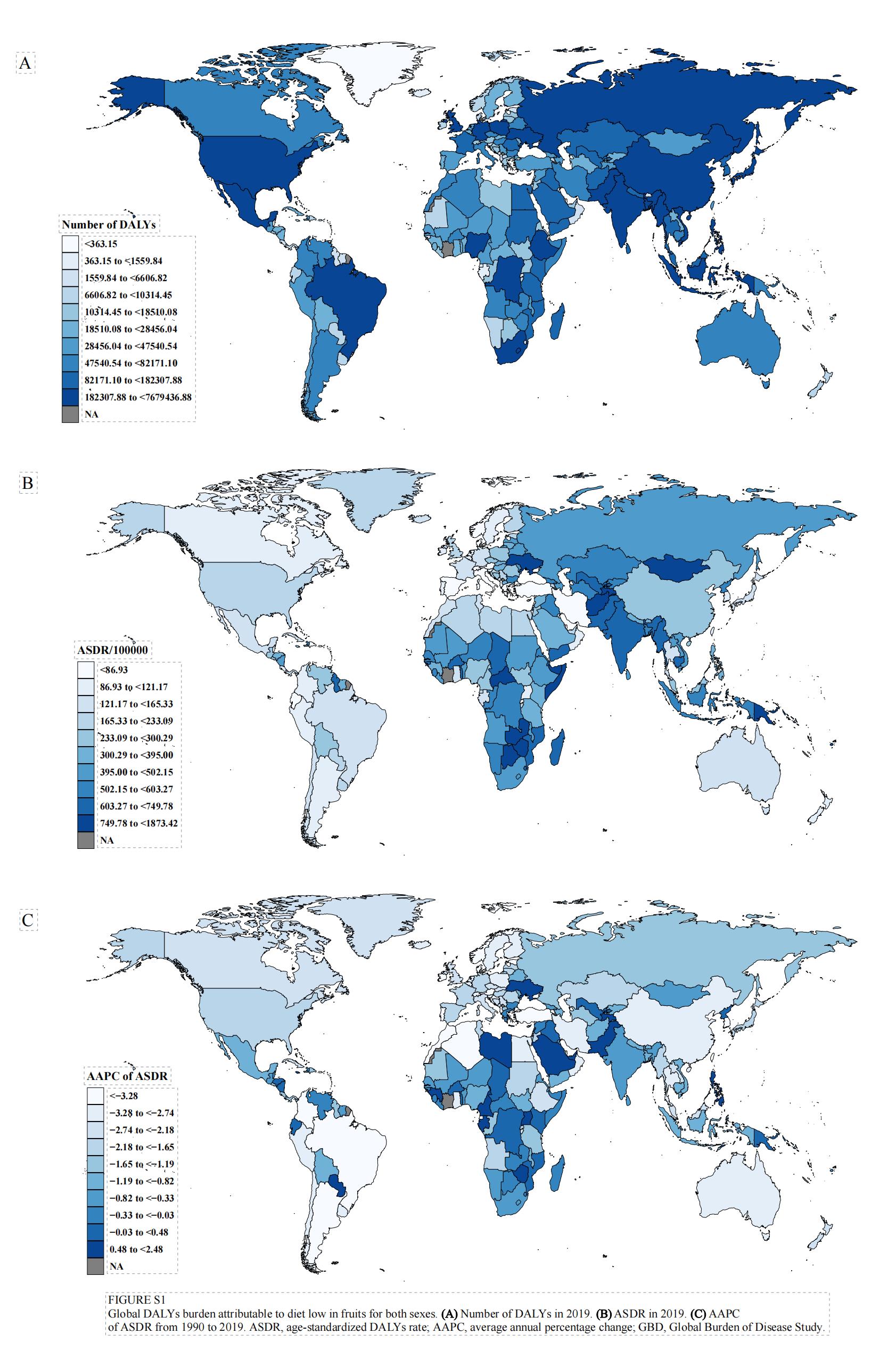

Supplement: Supplementary file 1 [file Image_1.JPEG]

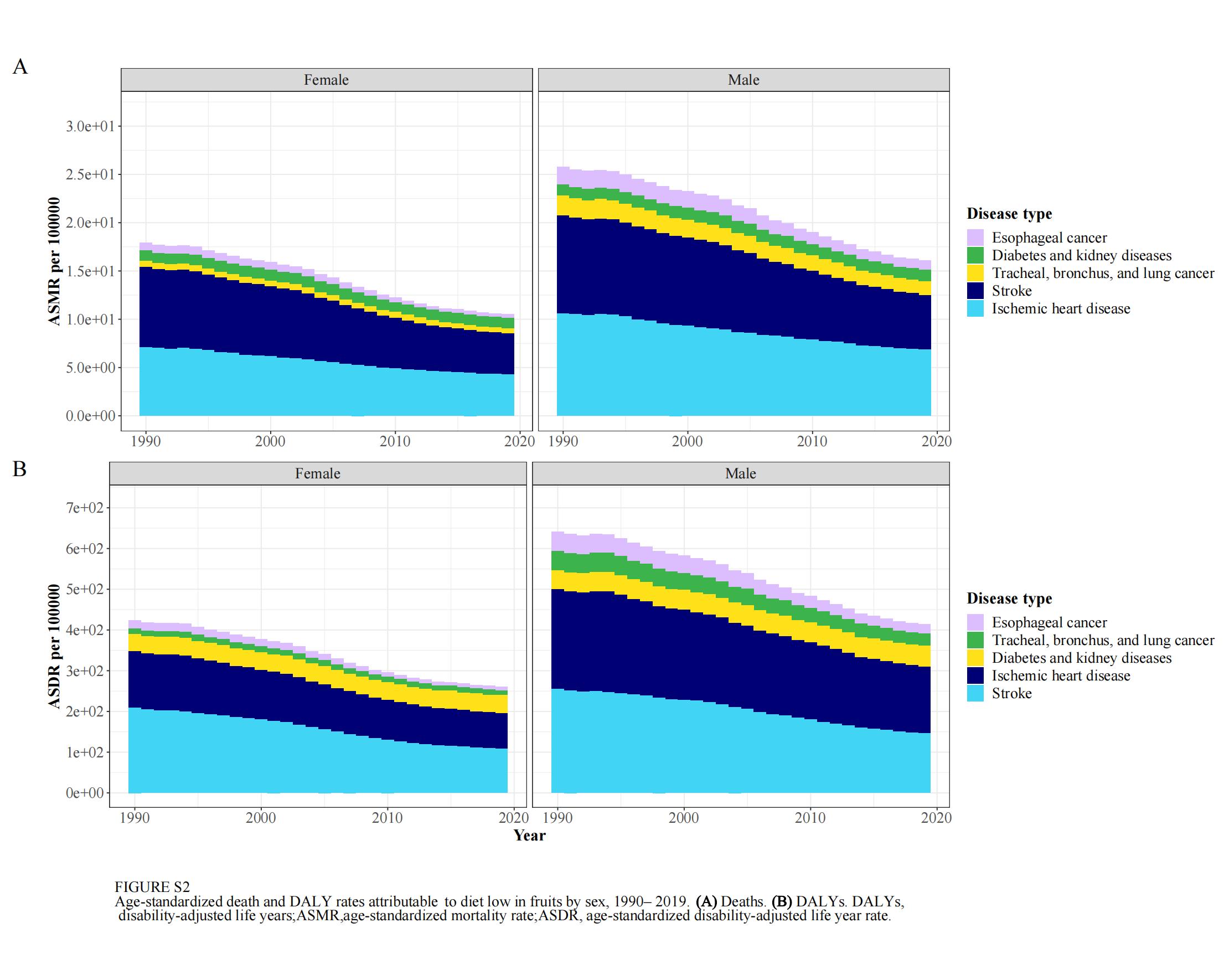

Supplement: Supplementary file 2 [file Image_2.JPEG]

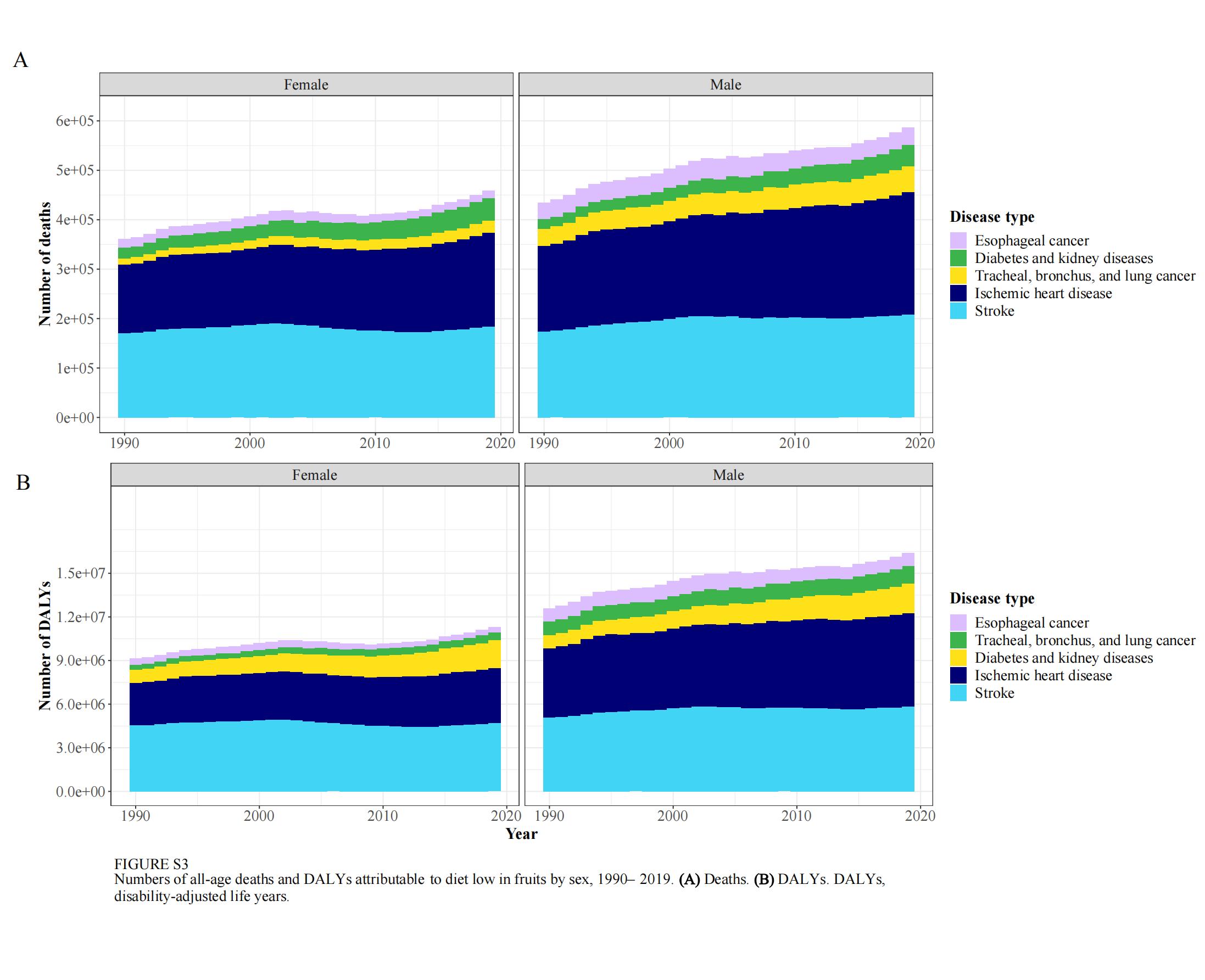

Supplement: Supplementary file 3 [file Image_3.JPEG]

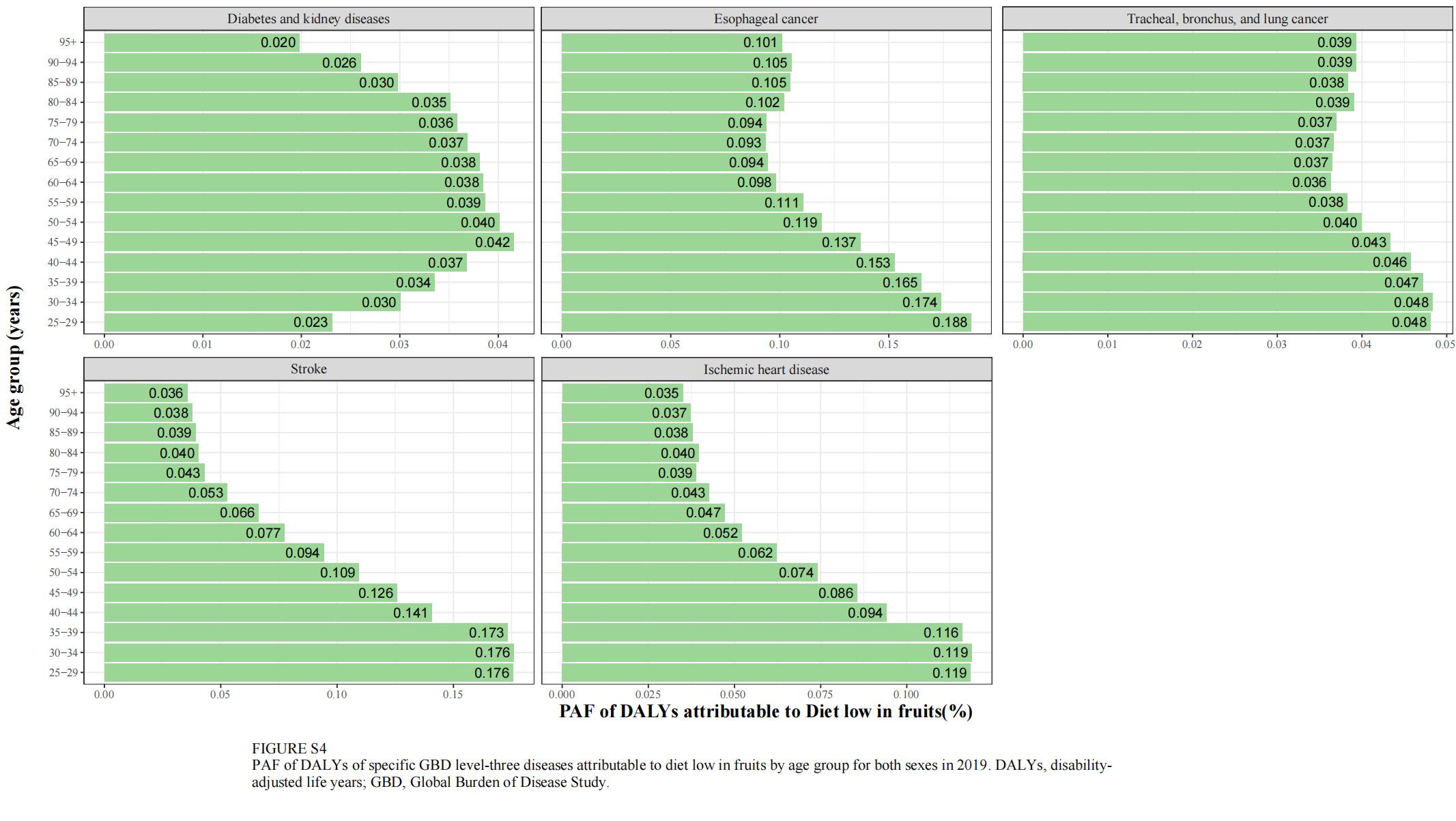

Supplement: Supplementary file 4 [file Image_4.JPEG]

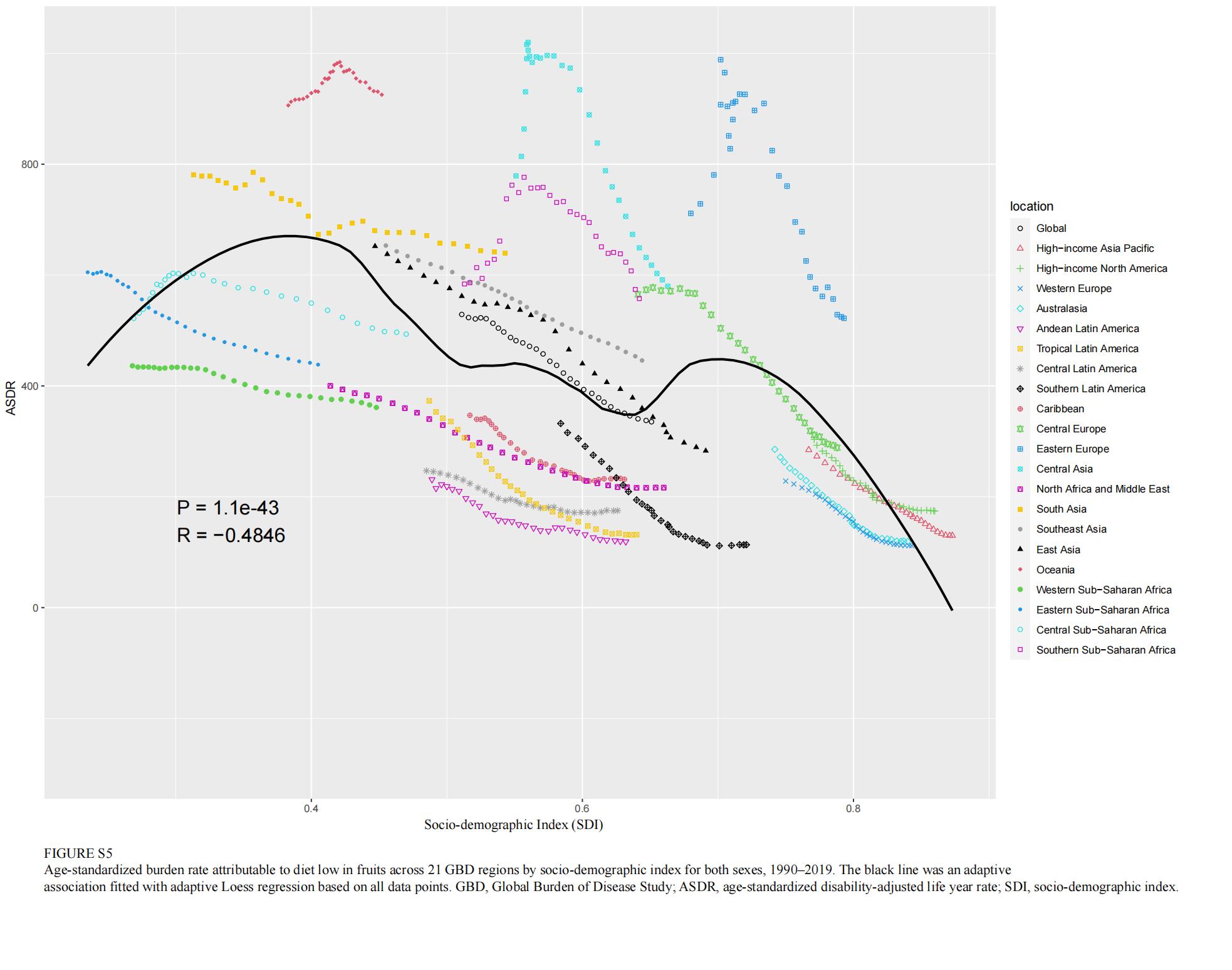

Supplement: Supplementary file 5 [file Image_5.JPEG]

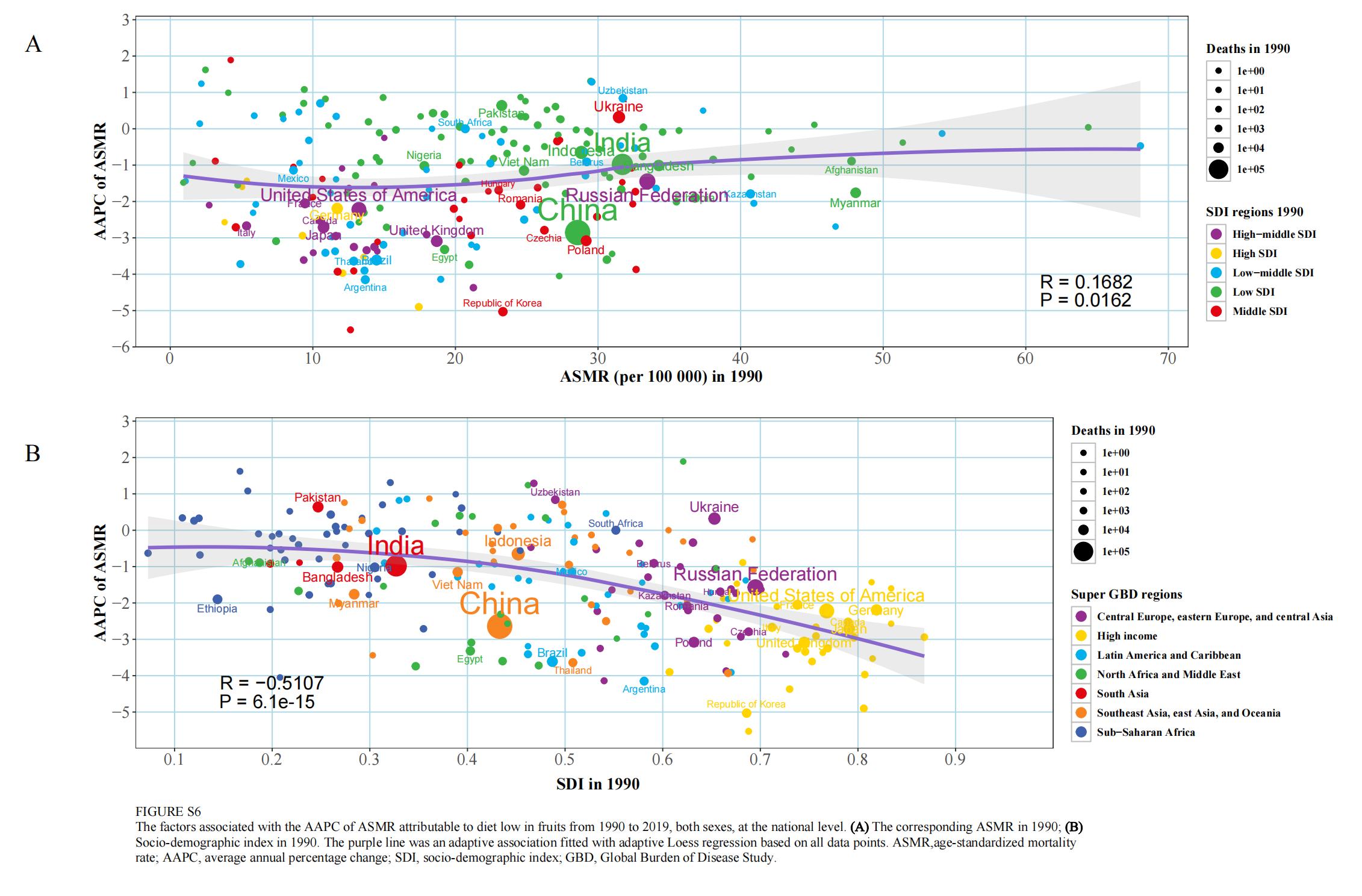

Supplement: Supplementary file 6 [file Image_6.JPEG]

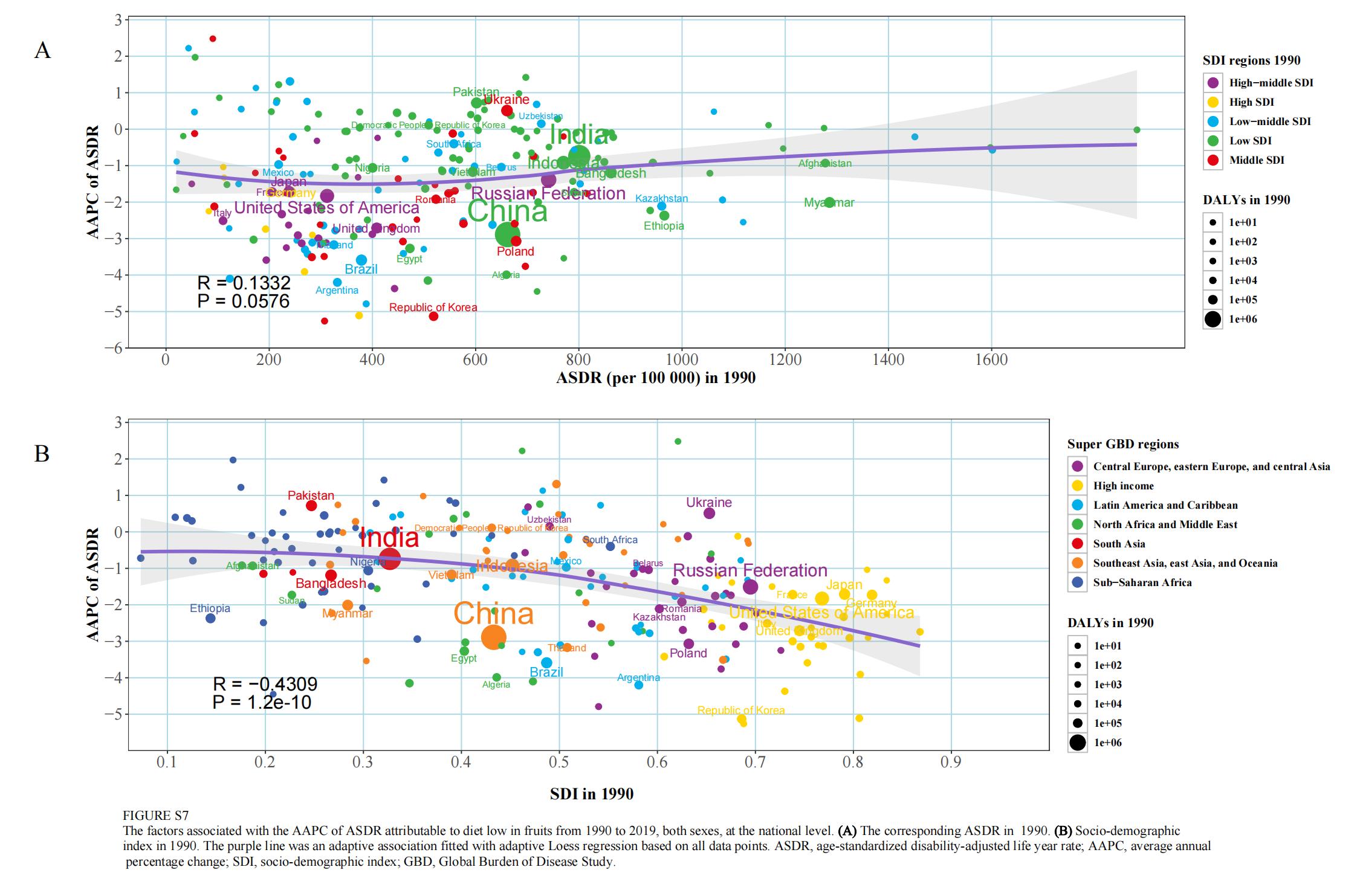

Supplement: Supplementary file 7 [file Image_7.JPEG]

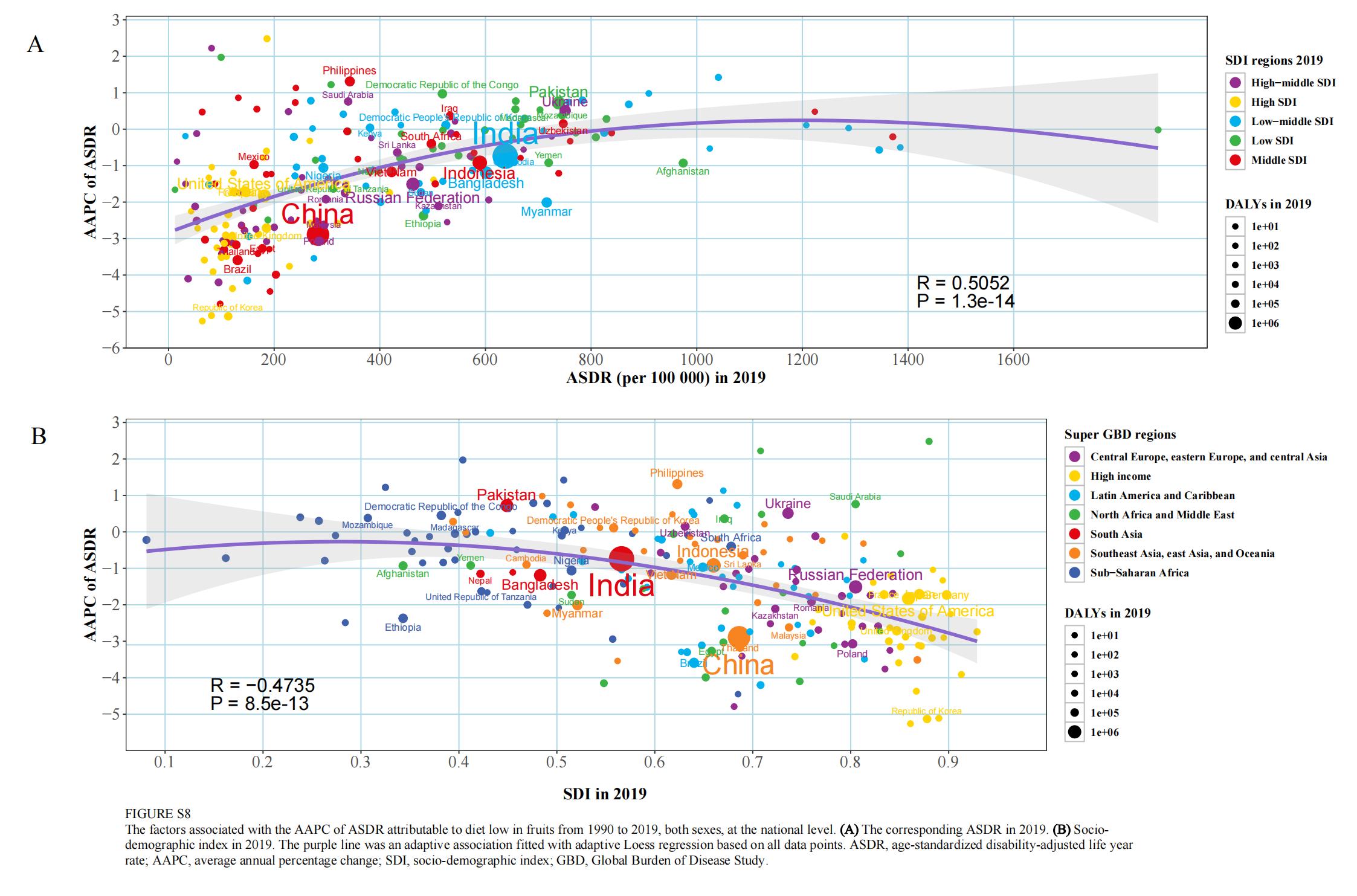

Supplement: Supplementary file 8 [file Image_8.JPEG]

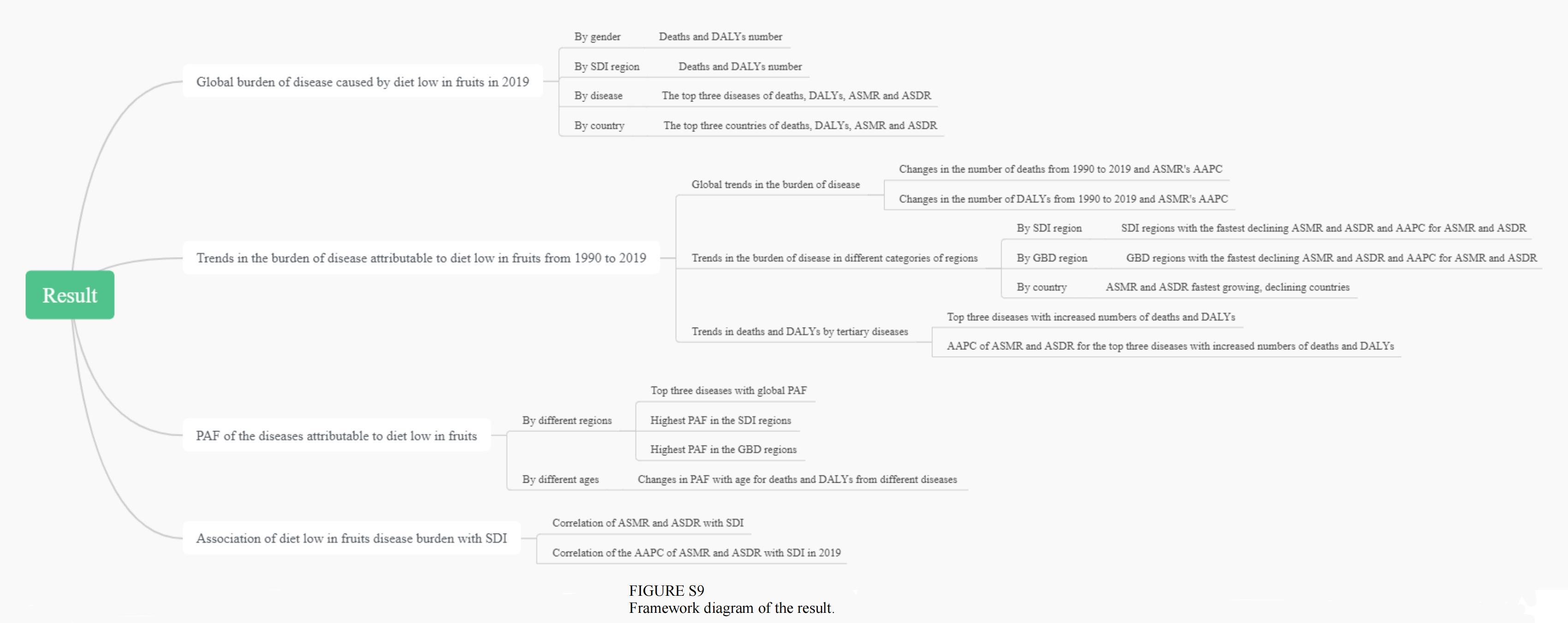

Supplement: Supplementary file 9 [file Image_9.JPEG]
