# Supplementary material for: Global burden of non-communicable chronic diseases associated with a diet low in fruits from 1990 to 2019
Source: Front Nutr. 2023 Aug 24;10:1202763. doi: 10.3389/fnut.2023.1202763 (PMC10491017; doi:10.3389/fnut.2023.1202763)
Supplement: Supplementary file 10 [file Table_1.DOCX]

| **Table S1.** Global DALYs attributable to diet low in fruits in 1990 and 2019, and the temporal trend from 1990 to 2019. | | | | | | | | | | |
| --- | --- | --- | --- | --- | --- | --- | --- | --- | --- | --- |
| **Cause of DALYs** | **1990** | | |  | **2019** | | |  | **1990-2019** | |
|  | **DALYs**  **No.×10^3^ (95% UI))** | **ASDR per 100 000**  **(95%UI)** | **Age-standardized**  **PAF, %**  **(95%UI)** |  | **DALYs**  **No.×10^3^ (95% UI))** | **ASDR per 100 000**  **(95%UI)** | **Age-standardized**  **PAF, %**  **(95%UI)** |  | **AAPC of**  **ASDR**  **(95%CI)** | **AAPC of**  **Age-standardized**  **PAF(95%CI)** |
| **All causes** |  |  |  |  |  |  |  |  |  |  |
| Both | 2172.16(1556.95,2819.96) | 528.92(377.77,685.95) | 1.06 (0.75,1.37) |  | 2767.83(2022.67,3592.54) | 335.28(244.44,434.87) | 1.02 (0.73,1.32) |  | -1.56(-1.69,-1.42) | -0.11(-0.26,0.04) |
| Female | 913.04 (652.21,1195.43) | 424.69(303.97,557.58) | 0.92 (0.65,1.18) |  | 1128.98 (825.67,1460.12) | 261 (191.15,337.38) | 0.86 (0.62,1.12) |  | -1.66(-1.75,-1.58) | -0.19(-0.34,-0.05) |
| Male | 1259.12 (897.82,1637.03) | 640.95(461.13,833.62) | 1.18 (0.83,1.53) |  | 1638.85(1178.77,2156.14) | 414.25(297.82,545.49) | 1.16 (0.82,1.51) |  | -1.49(-1.63,-1.34) | -0.06(-0.21,0.08) |
| **Disease type** |  |  |  |  |  |  |  |  |  |  |
| **Neoplasms** |  |  |  |  |  |  |  |  |  |  |
| Both | 260.48 (147.09,389.71) | 63.02 (35.45,94.35) | 1.65 (0.92,2.45) |  | 299.73 (154.42,467.97) | 35.98 (18.53,56.19) | 1.18 (0.57,1.81) |  | -1.93(-2.05,-1.81) | -1.18(-1.23,-1.13) |
| Female | 75.39 (42.63,113.77) | 34.86 (19.66,52.72) | 1.09 (0.61,1.67) |  | 90.22 (48.37,132.83) | 20.75 (11.15,30.53) | 0.76 (0.43,1.17) |  | -1.78(-1.91,-1.66) | -1.08(-1.18,-0.98) |
| Male | 185.09 (105.22,278.75) | 94.38 (53.35,141.93) | 2.05 (1.14,3.07) |  | 209.51 (104.97,335.6) | 52.85 (26.48,84.65) | 1.46 (0.73,2.31) |  | -2.00(-2.22,-1.78) | -1.19(-1.25,-1.12) |
| **Esophageal cancer** |  |  |  |  |  |  |  |  |  |  |
| Both | 135.85 (47.32,238.72) | 32.83 (11.36,57.97) | 16.48 (5.93,28.68) |  | 124.98 (38.45,259.51) | 14.96 (4.59,31.05) | 10.73 (3.41,22.58) |  | -2.71(-2.85,-2.57) | -1.47(-1.54,-1.41) |
| Female | 43.44 (16.06,76.16) | 20.12 (7.43,35.37) | 17.54 (6.74,29.59) |  | 35.73 (12.86,66.83) | 8.23 (2.98,15.36) | 12.64 (4.67,23.87) |  | -3.08(-3.32,-2.83) | -1.10(-1.24,-0.96) |
| Male | 92.41 (30.76,166.54) | 46.68 (15.36,83.83) | 15.98 (5.48,28.28) |  | 89.25 (26.29,192.79) | 22.31 (6.49,48.36) | 10.08 (2.93,22.13) |  | -2.53(-2.74,-2.33) | -1.57(-1.62,-1.53) |
| **Tracheal, bronchus, and lung cancer** |  |  |  |  |  |  |  |  |  |  |
| Both | 124.63 (41.73,185.61) | 30.18 (10.06,44.92) | 4.59 (1.51,6.81) |  | 174.75 (51.83,260.88) | 21.02 (6.22,31.38) | 3.81 (1.13,5.67) |  | -1.23(-1.36,-1.09) | -0.64(-0.66,-0.62) |
| Female | 31.95 (10.77,48.07) | 14.75 (4.96,22.18) | 4.73 (1.58,7.01) |  | 54.49 (16.19,81.23) | 12.51 (3.72,18.66) | 3.82 (1.11,5.69) |  | -0.57(-0.65,-0.48) | -0.73(-0.75,-0.72) |
| Male | 92.68 (30.82,138.47) | 47.71 (15.72,71.32) | 4.53 (1.48,6.72) |  | 120.26 (36.37,180.74) | 30.54 (9.17,45.87) | 3.78 (1.14,5.68) |  | -1.51(-1.68,-1.35) | -0.60(-0.62,-0.58) |
| **Cardiovascular diseases** |  |  |  |  |  |  |  |  |  |  |
| Both | 1733.24(1142.91,2372.98) | 422.28(276.97,578.17) | 5.96 (3.89,8.03) |  | 2073.47(1323.86,2849.88) | 251.67(160.26,345.36) | 5.17 (3.32,7.02) |  | -1.76(-1.89,-1.62) | -0.48(-0.53,-0.44) |
| Female | 747.37 (490.21,1016.32) | 347.98(227.93,474.01) | 5.77 (3.82,7.81) |  | 846.29 (543.54,1160.05) | 195.87(126.02,268.46) | 4.96 (3.22,6.66) |  | -1.95(-2.03,-1.86) | -0.51(-0.56,-0.47) |
| Male | 985.86 (642.53,1354.02) | 500.84(321.09,691.52) | 6.07 (3.97,8.23) |  | 1227.17 (772.05,1708.83) | 310.23(194.86,432.28) | 5.26 (3.32,7.28) |  | -1.62(-1.79,-1.46) | -0.46(-0.52,-0.40) |
| **Ischemic heart disease** |  |  |  |  |  |  |  |  |  |  |
| Both | 771.76 (334.29,1128.88) | 190.73 (81.66,279.84) | 6.07 (2.63,8.84) |  | 1023.34 (424.14,1512.34) | 124.65 (51.59,184.53) | 5.55 (2.29,8.15) |  | -1.44(-1.59,-1.29) | -0.31(-0.38,-0.23) |
| Female | 293.03 (126.05,426.67) | 138.62 (59.17,201.56) | 5.86 (2.48,8.53) |  | 378.12 (157.87,559.03) | 87.36 (36.55,129.22) | 5.33 (2.22,7.82) |  | -1.56(-1.72,-1.4) | -0.32(-0.37,-0.26) |
| Male | 478.73 (207.22,702.63) | 245.61(105.05,360.85) | 6.13 (2.63,8.96) |  | 645.22 (269.01,969.04) | 163.73 (68.50,246.54) | 5.64 (2.32,8.32) |  | -1.40(-1.60,-1.20) | -0.29(-0.36,-0.21) |
| **Stroke** |  |  |  |  |  |  |  |  |  |  |
| Both | 961.48 (581.38,1459.18) | 231.55(140.16,352.31) | 8.48 (5.16,12.86) |  | 1050.13 (624.21,1604.44) | 127.02 (75.36,194.17) | 7.18 (4.29,10.94) |  | -2.04(-2.12,-1.96) | -0.56(-0.62,-0.51) |
| Female | 454.35 (273.36,685.75) | 209.35(125.07,318.18) | 8.36 (5.05,12.66) |  | 468.18 (278.66,706.57) | 108.51 (64.68,163.58) | 7.09 (4.24,10.73) |  | -2.22(-2.29,-2.15) | -0.56(-0.64,-0.47) |
| Male | 507.13 (308.12,773.24) | 255.23(153.93,389.43) | 8.57 (5.18,12.96) |  | 581.95 (345.98,905.48) | 146.46 (86.83,227.91) | 7.24 (4.31,11.09) |  | -1.88(-1.97,-1.79) | -0.58(-0.64,-0.53) |
| **Diabetes and kidney diseases** |  |  |  |  |  |  |  |  |  |  |
| Both | 178.45 (111.22,257.43) | 43.62 (26.98,62.92) | 3.66 (2.35,5.21) |  | 394.63 (237.01,590.81) | 47.63 (28.63,71.34) | 3.45 (2.13,5.03) |  | 0.28(0.16,0.40) | -0.24(-0.31,-0.18) |
| Female | 90.28 (55.76,131.47) | 41.85 (25.85,61.14) | 3.66 (2.34,5.27) |  | 192.46 (116.52,287.61) | 44.38 (26.82,66.22) | 3.51 (2.17,5.12) |  | 0.19(0.08,0.31) | -0.22(-0.35,-0.09) |
| Male | 88.17 (55.09,126.41) | 45.71 (28.45,65.74) | 3.61 (2.33,5.11) |  | 202.17 (121.43,305.72) | 51.18 (30.67,77.4) | 3.39 (2.08,4.94) |  | 0.38(0.22,0.54) | -0.23(-0.35,-0.11) |
| **Diabetes mellitus** |  |  |  |  |  |  |  |  |  |  |
| Both | 178.45 (111.22,257.43) | 43.62 (26.98,62.92) | 6.31 (4.04,8.81) |  | 394.63 (237.01,590.81) | 47.63 (28.63,71.34) | 5.54 (3.36,7.97) |  | 0.28(0.16,0.40) | -0.46(-0.50,-0.43) |
| Female | 90.28 (55.76,131.48) | 41.85 (25.85,61.14) | 6.22 (3.99,8.73) |  | 192.46 (116.52,287.61) | 44.38 (26.82,66.22) | 5.57 (3.41,8.04) |  | 0.19(0.08,0.31) | -0.39(-0.47,-0.31) |
| Male | 88.17 (55.09,126.41) | 45.71 (28.45,65.74) | 6.41 (4.13,8.89) |  | 202.17 (121.43,305.72) | 51.18 (30.68,77.36) | 5.52 (3.39,7.94) |  | 0.38(0.22,0.54) | -0.52(-0.61,-0.43) |

1. ASDR, age-standardized DALYs rate;
2. PAF, population attributable fraction;
3. AAPC, average annual percentage change;
4. UI, uncertainty interval;
5. CI, confidence interval.
