# Supplementary material for: Global burden of non-communicable chronic diseases associated with a diet low in fruits from 1990 to 2019
Source: Front Nutr. 2023 Aug 24;10:1202763. doi: 10.3389/fnut.2023.1202763 (PMC10491017; doi:10.3389/fnut.2023.1202763)
Supplement: Supplementary file 11 [file Table_2.DOCX]

| **Table S2.** Deaths and ASMR in all causes attributed to diet low in fruits in 1990 and 2019, and its average annual percentage change from 1990 to 2019, by location. | | | | | | | | | | |
| --- | --- | --- | --- | --- | --- | --- | --- | --- | --- | --- |
| **Characteristics** | **1990** | | |  | **2019** | | |  | **1990-2019** | |
|  | **Deaths**  **No.×10^3^ (95% UI)** | **ASMR per 100000**  **No. (95% UI)** | **Age-standardized**  **PAF No./10^2^, %**  **(95%UI)** |  | **Deaths**  **No.×10^3^ (95% UI)** | **ASMR per 100000**  **No. (95% UI)** | **Age-standardized**  **PAF No./10^2^, %**  **(95%UI)** |  | **AAPC in ASMR**  **No. (95% CI)** | **AAPC of**  **Age-standardized**  **PAF(95%CI)** |
| **SDI region** |  |  |  |  |  |  |  |  |  |  |
| High SDI | 138.09 (85.75,183.86) | 13.38 (8.28,17.79) | 1.97 (1.22,2.62) |  | 122.76 (79.01,163.11) | 6.07 (3.94,8.05) | 1.36 (0.88,1.78) |  | -2.67(-2.85,-2.49) | -1.28(-1.31,-1.25) |
| High-middle SDI | 220.93(149.63,291.42) | 22.76(15.22,30.09) | 2.37 (1.58,3.08) |  | 228.62(149.56,302.33) | 11.51 (7.49,15.27) | 1.89 (1.22,2.51) |  | -2.31(-2.56,-2.05) | -0.76(-0.89,-0.63) |
| Middle SDI | 228.26(159.53,302.01) | 24.81(17.28,32.95) | 2.24 (1.58,2.97) |  | 325.58(226.46,434.28) | 14.36 (9.92,19.17) | 1.96 (1.35,2.58) |  | -1.87(-1.98,-1.76) | -0.47(-0.56,-0.38) |
| Low-middle SDI | 155.86 (112.65,200.83) | 28.02(20.41,36.29) | 1.91 (1.4,2.48) |  | 271.39(192.29,355.68) | 21.15 (15,27.62) | 2.23 (1.59,2.87) |  | -0.95(-1.08,-0.81) | 0.58(0.50,0.66) |
| Low SDI | 52.12 (36.58,69.26) | 23.89(16.87,31.86) | 1.33 (0.95,1.76) |  | 97.18 (68.89,129.58) | 20.32(14.39,27.03) | 1.78 (1.26,2.34) |  | -0.59(-0.64,-0.55) | 0.99(0.87,1.11) |
| **GBD region** |  |  |  |  |  |  |  |  |  |  |
| High-income Asia Pacific | 22.75 (15.56,30.16) | 12.42 (8.34,16.45) | 2.08 (1.39,2.76) |  | 23.84 (15.89,31.63) | 4.69 (3.28,6.15) | 1.38 (0.97,1.84) |  | -3.32(-3.49,-3.14) | -1.35(-1.43,-1.28) |
| High-income North America | 46.12 (26.89,62.37) | 12.97 (7.63,17.58) | 1.96 (1.15,2.65) |  | 43.82 (26.81,59.23) | 6.72 (4.13,9.03) | 1.29 (0.76,1.76) |  | -2.25(-2.40,-2.10) | -1.38(-1.43,-1.33) |
| Western Europe | 62.17 (38.18,84.08) | 10.82 (6.62,14.52) | 1.59 (0.98,2.14) |  | 47.86 (30.48,64.66) | 4.74 (3.12,6.26) | 1.12 (0.73,1.48) |  | -2.81(-3.00,-2.62) | -1.21(-1.27,-1.16) |
| Australasia | 3.08 (1.82,4.16) | 13.74 (8.05,18.59) | 2.12 (1.24,2.87) |  | 2.86 (1.78,3.91) | 5.35 (3.42,7.23) | 1.35 (0.86,1.83) |  | -3.16(-3.42,-2.91) | -1.53(-1.61,-1.45) |
| Southern Latin America | 6.13 (3.92,8.32) | 14.08 (8.93,19.19) | 1.72 (1.09,2.34) |  | 3.92 (2.51,5.46) | 4.66 (3.01,6.49) | 0.78 (0.46,1.08) |  | -3.73(-4.01,-3.46) | -2.71(-2.79,-2.62) |
| Andean Latin America | 1.84 (1.18,2.53) | 9.42 (6.02,12.89) | 1.02 (0.66,1.35) |  | 2.59 (1.58,3.73) | 4.71 (2.89,6.77) | 0.82 (0.53,1.11) |  | -2.34(-2.96,-1.72) | -0.67(-0.92,-0.42) |
| Tropical Latin America | 12.36 (8.09,16.88) | 14.23 (9.08,19.43) | 1.41 (0.86,1.93) |  | 11.84 (7.57,16.57) | 4.96 (3.15,7.02) | 0.79 (0.51,1.11) |  | -3.50(-3.67,-3.33) | -1.98(-2.05,-1.91) |
| Central Latin America | 7.63 (4.76,10.31) | 9.79 (6.05,13.29) | 1.14 (0.71,1.55) |  | 15.12 (9.44,21.47) | 6.56 (4.05,9.35) | 1.06 (0.65,1.48) |  | -1.41(-1.59,-1.22) | -0.28(-0.35,-0.21) |
| Caribbean | 3.47 (2.17,4.68) | 13.89 (8.61,18.7) | 1.47 (0.91,1.98) |  | 4.39 (2.87,6.12) | 8.47 (5.53,11.76) | 1.12 (0.74,1.52) |  | -1.64(-1.78,-1.49) | -1.30(-1.75,-0.85) |
| Eastern Europe | 82.96 (51.09,111.87) | 32.55(19.92,44.13) | 3.24 (1.99,4.36) |  | 79.87 (45.65,110.76) | 23.36(13.37,32.25) | 2.78 (1.58,3.81) |  | -1.02(-1.81,-0.23) | -0.52(-0.58,-0.45) |
| Central Europe | 33.92 (20.61,45.99) | 25.15(15.07,34.28) | 2.53 (1.51,3.45) |  | 27.64 (16.96,39.03) | 12.79 (7.85,18.02) | 1.97 (1.22,2.65) |  | -2.33(-2.59,-2.08) | -0.89(-0.97,-0.81) |
| Central Asia | 14.48 (9.37,19.18) | 33.23(21.43,44.22) | 3.22 (2.07,4.28) |  | 16.35 (9.83,22.72) | 26.51(15.59,36.98) | 2.66 (1.57,3.63) |  | -0.73(-0.98,-0.47) | -0.66(-0.81,-0.51) |
| North Africa and Middle East | 25.58 (14.4,36.19) | 16.25 (9.27,22.99) | 1.42 (0.81,1.99) |  | 34.59 (20.56,49.78) | 8.47 (4.97,12.25) | 1.09 (0.63,1.55) |  | -2.22(-2.42,-2.02) | -0.91(-1.09,-0.74) |
| South Asia | 158.6 (111.81,201.79) | 30.71(21.83,39.15) | 1.99 (1.42,2.56) |  | 318.57(221.63,418.01) | 24.29(16.72,31.87) | 2.65 (1.84,3.39) |  | -0.84(-1.00,-0.68) | 1.04(0.93,1.15) |
| Southeast Asia | 60.43 (42.48,81.28) | 25.26(17.94,33.93) | 2.15 (1.58,2.98) |  | 98.29 (69.24,133.35) | 17.33(12.13,23.34) | 2.14 (1.51,2.87) |  | -1.27(-1.32,-1.23) | -0.11(-0.19,-0.02) |
| East Asia | 211.88(143.31,292.06) | 28.07(19.01,38.49) | 2.51 (1.71,3.43) |  | 238.83(155.59,334.16) | 13.05 (8.45,18.38) | 2.06 (1.38,2.84) |  | -2.61(-2.79,-2.43) | -0.67(-0.74,-0.60) |
| Oceania | 0.94 (0.65,1.28) | 32.76 (22.79,44.73) | 2.36 (1.71,3.17) |  | 2.26 (1.49,3.16) | 33.05 (22.52,45.46) | 2.63 (1.87,3.43) |  | 0.02(-0.01,0.06) | 0.32(0.25,0.38) |
| Western Sub-Saharan Africa | 14.51 (9.87,20.21) | 18.82 (12.89,26.06) | 1.13 (0.78,1.56) |  | 25.61 (17.81,34.83) | 15.63 (10.9,20.91) | 1.31 (0.92,1.74) |  | -0.61(-0.66,-0.56) | 0.52(0.47,0.57) |
| Eastern Sub-Saharan Africa | 16.57 (12.02,22.05) | 23.48 (17.16,31) | 1.15 (0.87,1.58) |  | 26.62 (18.83,35.86) | 18.11 (12.87,24.37) | 1.56 (1.13,2.09) |  | -0.88(-0.98,-0.78) | 1.02(0.79,1.26) |
| Central Sub-Saharan Africa | 4.23 (2.82,6.02) | 20.79 (13.94,29.32) | 1.11 (0.76,1.55) |  | 9.39 (6.29,13.36) | 19.87 (13.42,28.04) | 1.59 (1.05,2.14) |  | -0.17(-0.31,-0.03) | 1.21(1.12,1.31) |
| Southern Sub-Saharan Africa | 5.88 (4.48,7.35) | 22.47 (17.1,27.95) | 1.94 (1.48,2.41) |  | 11.71 (8.97,14.58) | 23.04 (17.55,28.57) | 1.89 (1.45,2.33) |  | 0.09(-0.48,0.66) | -0.10(-0.35,0.15) |
| 1. No., number; 2. ASMR, age-standardized mortality rate; 3. UI, uncertainty interval; 4. AAPC, average annual percentage change; 5. CI, confidential interval;   [6] PAF, the proportion of cases of depression that could be prevented if the influence of all causes attributed to diet low in fruits was removed. | | | | | | | | | | |
