# Supplementary material for: Global burden of non-communicable chronic diseases associated with a diet low in fruits from 1990 to 2019
Source: Front Nutr. 2023 Aug 24;10:1202763. doi: 10.3389/fnut.2023.1202763 (PMC10491017; doi:10.3389/fnut.2023.1202763)
Supplement: Supplementary file 12 [file Table_3.DOCX]

| **Table S3.** DALYs and ASDR in all causes attributed to diet low in fruits in 1990 and 2019, and its average annual percentage change from 1990 to 2019, by location. | | | | | | | | | | |
| --- | --- | --- | --- | --- | --- | --- | --- | --- | --- | --- |
| **Characteristics** | **1990** | | |  | **2019** | | |  | **1990-2019** | |
|  | **DALYs（Deaths）**  **No.×10^4^ (95% UI)** | **ASDR per 100 000**  **No. (95% UI)** | **Age-standardized**  **PAF No./10^3^, %**  **(95%UI)** |  | **DALYs**  **No.×10^4^ (95% UI)** | **ASDR per 100 000**  **No. (95% UI)** | **Age-standardized**  **PAF No./10^3^, %**  **(95%UI)** |  | **AAPC in ASDR**  **No. (95% CI)** | **AAPC of**  **Age-standardized**  **PAF(95%CI)** |
| **SDI region** |  |  |  |  |  |  |  |  |  |  |
| High SDI | 304.32(200.78,398.03) | 300.98(199.47,394.89) | 11.08(7.17,14.79) |  | 265.79(181.26,353.57) | 155.72(106.13,206.94) | 7.35(5.01,9.75) |  | -2.25(-2.31,-2.19) | -1.40(-1.43,-1.37) |
| High-middle SDI | 563.24(396.56,736.34) | 524.56(367.08,685.01) | 14.32(9.87,18.77) |  | 529.59(368.48,695.38) | 265.84(184.22,349.86) | 11.12(7.64,14.69) |  | -2.29(-2.57,-2.02) | -0.85(-1.06,-0.63) |
| Middle SDI | 665.09(476.32,874.93) | 601.67(431.47,792.54) | 13.45(9.59,17.68) |  | 876.18(630.07,1158.47) | 344.97(247.25,458.13) | 12.01(8.45,15.92) |  | -1.91(-2.01,-1.81) | -0.40(-0.47,-0.33) |
| Low-middle SDI | 477.76(346.44,611.73) | 721.35(528.57,919.52) | 11.11(8.05,14.25) |  | 794.94(576.47,1028.67) | 549.82(398.47,710.32) | 13.96(10.11,17.85) |  | -0.92(-1.02,-0.82) | 0.84(0.74,0.93) |
| Low SDI | 160.81(115.28,212.03) | 611.21(438.23,803.83) | 7.27(5.18,9.56) |  | 299.93(215.82,395.32) | 519.26(376.11,685.57) | 10.54(7.61,13.59) |  | -0.58(-0.65,-0.51) | 1.31(1.19,1.43) |
| **GBD region** |  |  |  |  |  |  |  |  |  |  |
| High-income Asia Pacific | 56.69(40.21,74.63) | 284.64(201.69,375.05) | 12.23(8.47,16.45) |  | 49.47(36.01,64.67) | 129.95(96.21,169.57) | 8.07(5.82,10.49) |  | -2.67(-2.78,-2.56) | -1.42(-1.55,-1.29) |
| High-income North America | 102.23(63.03,135.97) | 303.56(188.16,404.26) | 10.55(6.47,14.19) |  | 100.23(66.12,135.82) | 174.12(115.27,235.23) | 6.87(4.48,9.24) |  | -1.88(-1.98,-1.78) | -1.46(-1.53,-1.39) |
| Western Europe | 124.97(81.09,166.01) | 228.22(147.99,302.76) | 8.79(5.58,11.84) |  | 91.88(62.94,121.47) | 111.74(77.43,147.88) | 5.82(3.87,7.74) |  | -2.46(-2.63,-2.29) | -1.42(-1.49,-1.35) |
| Australasia | 6.58(4.02,8.74) | 285.52(174.44,378.56) | 10.83(6.55,14.78) |  | 5.56(3.78,7.37) | 119.22(81.09,157.35) | 6.06(4,78.11.95) |  | -2.95(-3.08,-2.83) | -1.99(-2.12,-1.87) |
| Southern Latin America | 15.28(9.98,21.01) | 332.22(217.34,456.12) | 10.39(6.68,14.38) |  | 9.14(6.05,12.57) | 113.38(74.83,156.64) | 4.65(3.07,6.43) |  | -3.65(-3.87,-3.43) | -2.72(-2.84,-2.61) |
| Andean Latin America | 5.28(3.53,7.23) | 231.08(155.46,316.75) | 5.27(3.52,7.14) |  | 6.89(4.24,9.99) | 118.86(73.25,171.44) | 4.64(3.03,6.43) |  | -2.21(-2.81,-1.61) | -0.32(-0.70,0.06) |
| Tropical Latin America | 38.27(25.74,52.93) | 373.03(247.76,510.71) | 8.21(5.46,11.28) |  | 32.54(20.88,45.62) | 131.64(84.47,184.69) | 4.47(2.87,6.29) |  | -3.52(-3.66,-3.38) | -2.05(-2.19,-1.91) |
| Central Latin America | 22.86(14.97,31.03) | 246.89(160.51,336.42) | 6.54(4.24,8.98) |  | 42.38(26.78,60.01) | 174.96(109.94,248.15) | 6.37(4.02,8.88) |  | -1.18(-1.34,-1.02) | -0.08(-0.27,0.12) |
| Caribbean | 9.32(6.03,12.68) | 346.99(224.65,472.24) | 8.03(5.16,10.93) |  | 11.91(7.82,16.54) | 231.47(152.33,321.01) | 6.65(4.44,9.02) |  | -1.35(-1.46,-1.23) | -1.04(-1.59,-0.49) |
| Eastern Europe | 193.27(123.59,256.91) | 711.29(454.85,945.61) | 19.04(12.31,25.52) |  | 170.75(104.04,230.67) | 522.02(320.73,704.98) | 16.45(10.22,22.25) |  | -0.94(-1.83,-0.05) | -0.45(-0.72,-0.17) |
| Central Europe | 80.88(52.06,108.34) | 565.15(362.03,758.74) | 16.08(10.14,21.81) |  | 57.62(38.29,78.47) | 288.57(192.97,393.51) | 12.06(8.07,16.13) |  | -2.32(-2.54,-2.10) | -1.01(-1.08,-0.94) |
| Central Asia | 37.39(24.92,49.16) | 778.87(518.96,1024.62) | 18.26(12.07,23.98) |  | 44.05(28.08,60.75) | 579.81(364.12,795.12) | 16.81(10.51,22.97) |  | -1.01(-1.30,-0.72) | -0.26(-0.42,-0.11) |
| North Africa and Middle East | 76.41(45.07,106.77) | 400.07(237.27,557.54) | 8.11(4.82,11.31) |  | 107.19(66.84,151.92) | 216.56(133.97,306.45) | 6.96(4.32,9.71) |  | -2.09(-2.19,-1.99) | -0.53(-0.72,-0.34) |
| South Asia | 494.76(354.21,624.47) | 780.57(562.28,986.77) | 11.84(8.42,15.07) |  | 953.53(678.97,1225.57) | 639.39(456.64,820.65) | 16.56(11.92,20.98) |  | -0.68(-1.05,-0.32) | 1.22(0.98,1.46) |
| Southeast Asia | 186.71(132.72,250.41) | 653.27(465.54,871.99) | 13.33(9.46,17.65) |  | 289.48(205.65,392.43) | 445.95(317.74,602.22) | 14.18(10.08,19.16) |  | -1.29(-1.32,-1.25) | 0.22(0.11,0.33) |
| East Asia | 595.57(407.95,815.38) | 652.03(447.22,887.02) | 15.94(10.95,21.65) |  | 570.52(382.85,794.53) | 282.89(189.58,393.59) | 12.68(8.54,17.47) |  | -2.84(-2.99,-2.69) | -0.79(-0.89,-0.69) |
| Oceania | 3.22(2.24,4.38) | 906.08(637.47,1239.54) | 16.64(11.88,21.95) |  | 7.91(5.37,10.92) | 925.35(638.64,1264.47) | 18.76(13.45,24.59) |  | 0.09(0.05,0.13) | 0.39(0.35,0.42) |
| Western Sub-Saharan Africa | 40.55(27.93,56.77) | 436.26(303.13,606.22) | 5.31(3.68,7.37) |  | 74.32(52.72,101.44) | 361.05(257.92,488.56) | 6.75(4.88,8.99) |  | -0.65(-0.73,-0.56) | 0.83(0.78,0.88) |
| Eastern Sub-Saharan Africa | 51.04(36.95,67.47) | 605.08(445.44,798.29) | 6.64(4.83,8.76) |  | 80.17(57.76,107.65) | 438.39(316.31,586.56) | 9.12(6.58,11.99) |  | -1.12(-1.21,-1.02) | 1.19(0.94，1.43) |
| Central Sub-Saharan Africa | 13.23(8.89,18.67) | 521.92(351.38,734.85) | 6.01(4.11,8.34) |  | 29.51(20.22,41.84) | 493.47(344.13,699.56) | 9.61(6.76,12.81) |  | -0.20(-0.36,-0.04) | 1.60(1.39，1.81) |
| Southern Sub-Saharan Africa | 17.61(13.66,22.01) | 583.89(455.33,726.38) | 10.76(8.34,13.38) |  | 32.75(25.38,40.85) | 557.56(434.25,694.92) | 10.58(8.28,13.04) |  | -0.15(-0.72,0.41) | -0.08(-0.45,0.29) |
| 1. No., number; 2. ASDR, age-standardized DALYs rate; 3. UI, uncertainty interval; 4. AAPC, average annual percentage change; 5. CI, confidential interval;   [6] PAF, the proportion of cases of depression that could be prevented if the influence of all causes attributed to diet low in fruits was removed. | | | | | | | | | | |
